# Effect of Text Messaging and Behavioral Interventions on COVID-19 Vaccination Uptake: A Randomized Clinical Trial

**DOI:** 10.1001/jamanetworkopen.2022.16649

**Published:** 2022-06-13

**Authors:** Shivan J. Mehta, Colleen Mallozzi, Pamela A. Shaw, Catherine Reitz, Caitlin McDonald, Matthew Vandertuyn, Mohan Balachandran, Michael Kopinsky, Christianne Sevinc, Aaron Johnson, Robin Ward, Sae-Hwan Park, Christopher K. Snider, Roy Rosin, David A. Asch

**Affiliations:** 1 Department of Medicine, Perelman School of Medicine, University of Pennsylvania, Philadelphia; 2 Center for Health Care Innovation, University of Pennsylvania, Philadelphia; 3 Penn Medicine, University of Pennsylvania, Philadelphia; 4 Department of Biostatistics, Epidemiology, and Informatics, University of Pennsylvania, Philadelphia

## Abstract

**Importance:**

COVID-19 vaccine uptake among urban populations remains low.

**Objective:**

To evaluate whether text messaging with outbound or inbound scheduling and behaviorally informed content might increase COVID-19 vaccine uptake.

**Design, Setting, and Participants:**

This randomized clinical trial with a factorial design was conducted from April 29 to July 6, 2021, in an urban academic health system. The trial comprised 16 045 patients at least 18 years of age in Philadelphia, Pennsylvania, with at least 1 primary care visit in the past 5 years, or a future scheduled primary care visit within the next 3 months, who were unresponsive to prior outreach. The study was prespecified in the trial protocol, and data were obtained from the intent-to-treat population.

**Interventions:**

Eligible patients were randomly assigned in a 1:20:20 ratio to (1) outbound telephone call only by call center, (2) text message and outbound telephone call by call center to those who respond, or (3) text message, with patients instructed to make an inbound telephone call to a hotline. Patients in groups 2 and 3 were concurrently randomly assigned in a 1:1:1:1 ratio to receive different content: standard messaging, clinician endorsement (eg, “Dr. XXX recommends”), scarcity (“limited supply available”), or endowment framing (“We have reserved a COVID-19 vaccine appointment for you”).

**Main Outcomes and Measures:**

The primary outcome was the proportion of patients who completed the first dose of the COVID-19 vaccine within 1 month, according to the electronic health record. Secondary outcomes were the completion of the first dose within 2 months and completion of the vaccination series within 2 months of initial outreach. Additional outcomes included the percentage of patients with invalid cell phone numbers (wrong number or nontextable), no response to text messaging, the percentage of patients scheduled for the vaccine, text message responses, and the number of telephone calls made by the access center. Analysis was on an intention-to-treat basis.

**Results:**

Among the 16 045 patients included, the mean (SD) age was 36.9 (11.1) years; 9418 (58.7%) were women; 12 869 (80.2%) had commercial insurance, and 2283 (14.2%) were insured by Medicaid; 8345 (52.0%) were White, 4706 (29.3%) were Black, and 967 (6.0%) were Hispanic or Latino. At 1 month, 14 of 390 patients (3.6% [95% CI, 1.7%-5.4%]) in the outbound telephone call–only group completed 1 vaccine dose, as did 243 of 7890 patients (3.1% [95% CI, 2.7%-3.5%]) in the text plus outbound call group (absolute difference, −0.5% [95% CI, −2.4% to 1.4%]; *P* = .57) and 253 of 7765 patients (3.3% [95% CI, 2.9%-3.7%]) in the text plus inbound call group (absolute difference, −0.3% [95% CI, −2.2% to 1.6%]; *P* = .72). Among the 15 655 patients receiving text messaging, 118 of 3889 patients (3.0% [95% CI, 2.5%-3.6%]) in the standard messaging group completed 1 vaccine dose, as did 135 of 3920 patients (3.4% [95% CI, 2.9%-4.0%]) in the clinician endorsement group (absolute difference, 0.4% [95% CI, −0.4% to 1.2%]; *P* = .31), 100 of 3911 patients (2.6% [95% CI, 2.1%-3.1%]) in the scarcity group (absolute difference, −0.5% [95% CI, −1.2% to 0.3%]; *P* = .20), and 143 of 3935 patients (3.6% [95% CI, 3.0%-4.2%]) in the endowment group (absolute difference, 0.6% [95% CI, −0.2% to 1.4%]; *P* = .14).

**Conclusions and Relevance:**

There was no detectable increase in vaccination uptake among patients receiving text messaging compared with telephone calls only or behaviorally informed message content.

**Trial Registration:**

ClinicalTrials.gov Identifier: NCT04834726

## Key Points

**Question:**

Can text messaging with behavioral insights increase participation in COVID-19 vaccine outreach?

**Findings:**

In this randomized clinical trial comprising 16 045 participants, text messaging did not result in a higher response rate than outbound telephone calls. Behaviorally informed messaging did not result in a significantly higher response than usual content.

**Meaning:**

Text messaging offers a low-cost alternative to outbound telephone calls, but additional efforts are needed to increase vaccine uptake.

## Teaser

This randomized clinical trial evaluates whether text messaging with outbound or inbound scheduling and behaviorally informed content might increase COVID-19 vaccine uptake.

## Introduction

Despite considerable public health efforts to increase rates of COVID-19 vaccination, uptake remains limited. Lower vaccination rates among some racial and ethnic and sociodemographic groups exacerbate existing disparities in COVID-19 infection and its outcomes.^1^

Challenges of vaccine acceptance occur alongside logistical challenges of large-scale equitable vaccine outreach. In the initial phases of vaccine outreach, health systems and the government often relied on complex scheduling processes that required internet access and smartphones. Prior to this trial, our health system relied on emails and the patient portal within our electronic health record (EHR) to offer the vaccine to a large population of patients, but the experience revealed racial and ethnic and socioeconomic differences in uptake.

We aimed to overcome these challenges with 2 approaches. One was to switch to text messaging, which has facilitated more efficient and equitable outreach by health systems, as most patients have and use a telephone with texting capabilities.^2,3^ The second approach was to deploy insights from the field of behavioral science, as humans have systematic biases or heuristics that impede participation in health prevention activities.^4,5,6^ However, these same biases can often be overcome or even harnessed with different message frames that increase uptake of screening and vaccination.^7,8,9^ The concept of endowment suggests that people place higher value on items in their possession. If the vaccine is described as already reserved for the patient, that approach could take advantage of this sense of ownership, as patients may feel remorse giving up the allocated vaccine.^7,10,11,12^ Humans are also motivated by a sense of scarcity, placing more value on objects that are less readily available, which was the situation in the earlier phases of rollout of the COVID-19 vaccine.^13,14^ Patients may also trust the recommendation of their primary care clinician to overcome hesitance and receive the vaccine by leveraging the behavioral principle of social norms.^15,16,17^ Finally, reducing logistical barriers or the number of steps in the process to schedule a COVID-19 vaccine might make it easier for patients to receive the vaccine. For example, pairing text messaging with outbound telephone calls by the call center to patients reduces the effort by patients but also requires that the patient be available to answer, while an inbound telephone hotline allows patients to call directly at a convenient time as well as providing a sense of exclusivity. We conducted a randomized clinical trial among residents in the city of Philadelphia, a larger city with a diverse population, providing outreach to unresponsive and previously unavailable patients, and concurrently evaluated text messaging and scheduling workflows with messaging informed by behavioral science principles.

## Methods

### Study Design

This randomized clinical trial with a factorial design was conducted from April 29 to July 6, 2021. Patients were randomly assigned in a 1:20:20 ratio to 3 study groups related to scheduling modality workflow: (1) an outbound telephone call group (outbound telephone only group), (2) a text message group allowing patients to send back a text that results in an outbound telephone call (text plus outbound call group), or (3) a text messaging group allowing patients to call a direct scheduling telephone line (text plus inbound call group). Among the patients receiving text messaging (groups 2 and 3), patients were additionally randomly assigned in a 1:1:1:1 ratio in a factorial design to 4 different types of messaging content, informed by principles of behavioral science: standard messaging, clinician endorsement, scarcity, and endowment. These behavioral principles were identified through preliminary work done by our team to understand patient motivations. The study was approved by the institutional review board at the University of Pennsylvania. A waiver of informed consent was obtained because the study posed minimal risk to patients, was embedded in clinical operations, and could not have been practicably carried out without the waiver.^18^ The trial protocol and statistical analysis plan appear in Supplement 1. This trial followed the Consolidated Standards of Reporting Trials (CONSORT) reporting guideline for randomized studies.

The major changes to the protocol before trial launch include expansion of the inclusion criteria to all adults, altering group 3 to allow for patients to call a scheduling hotline, and reducing the number of patients in group 1 to accommodate limited call center capacity. There were no major changes to the protocol after trial recruitment.

### Study Population

Using our EHR, we identified all patients at least 18 years of age residing in Philadelphia, Pennsylvania, with at least 1 visit with a primary care clinician in the past 5 years, or a future scheduled visit with a primary care clinician within the next 3 months, on file at the University of Pennsylvania Health System. We excluded patients who had completed any dose of the COVID-19 vaccine at our health system, were currently scheduled for any dose of the vaccine, had any external documentation of vaccination through databases made available through our EHR, or had previously received text message–based vaccine outreach. All of the patients who had electronic patient portal accounts had received outreach prior to this trial (patient portal message with option for online scheduling and an additional email for those with a recorded address) and were eligible for this study.

### Interventions

Eligible patients were randomly assigned in permuted blocks to 3 study groups in a 1:20:20 ratio to receive outbound telephone call only, text message plus outbound telephone call, or text message plus inbound telephone call. This ratio was selected to have a comparable number of outbound telephone calls made by the call center in groups 1 and 2, and to avoid overburdening the call center. Randomization and text messaging were conducted using the Way to Health platform, a National Institutes of Health–funded software platform that facilitates and automates many aspects of study design and intervention implementation.^19^ Patients did not need to have an account with Way to Health to participate. Patients in group 1 received a telephone call to schedule their appointment from an access center representative at the health system. Access center representatives made up to 3 telephone call attempts. Patients randomly assigned to this group did not receive any text messaging.

All eligible patients randomized to the text message groups received text messaging about the COVID-19 vaccine from our health system. The first message (sent at 9:30 am) introduced the vaccine outreach and required the patient to confirm their name (“Hello, this is Penn Medicine reaching out to you about your interest in the COVID-19 vaccine. Can you confirm you are XXX?”). It also allowed for patients to opt out of further outreach (by replying with a text message of “STOP”). This was required for privacy reasons to allow us to send text message outreach. If the patient confirmed their name by replying “YES,” the second text message described that the patient was eligible for the COVID-19 vaccine (“Our records show you are eligible for your COVID-19 vaccine at Penn Medicine.”). This message was followed by a behaviorally informed messaging prompt, followed by the mechanism through which the patient can schedule the vaccine (described below for each group). At the end of the message, there was also a prompt to receive more information (by replying with a text message of “INFO”), to defer the vaccine for now (by replying with a text message of “NOT NOW”), or to indicate that vaccination had already been completed (by replying with a text message of “DONE”).

Patients in group 2 received text messaging that included a prompt to agree to schedule the vaccine: “If you would like us to call you to schedule your appointment please reply, YES.” A list of patients who replied “YES” was forwarded to the access center, where a representative made up to 3 attempts to schedule an appointment via a telephone call.

Patients in group 3 received text messaging that included a prompt to agree to schedule the vaccine, which was followed by a prompt to call the health system COVID-19 vaccine scheduling hotline along with the scheduling telephone number (Monday through Friday, 8 am-5 pm). Patients in this group had to opt in with a text message of “YES” to receive the vaccine scheduling hotline number, and were then directed to call to schedule the vaccine themselves.

Within text messaging groups 2 and 3, patients were concurrently randomized in a factorial design in permuted blocks to receive different messaging content informed by principles of behavioral science in a 1:1:1:1 ratio. Standard messaging said, “Our records show you are eligible for your COVID-19 vaccine at Penn Medicine.” Clinician endorsement from a primary care clinician said, “Dr. XXX recommends that you receive the vaccination.” A scarcity-based message said, “You have been selected to receive from the limited supply of COVID-19 vaccine at Penn Medicine.” An endowment-based message said, “We have reserved a COVID-19 vaccine appointment for you at Penn Medicine.” Patients were messaged in 5 batches (in permuted blocks by study group) distributed over 5 business days between April 28 and May 5, 2021, to avoid overwhelming the access center. For all text message study groups, patients received up to 2 reminder text messages at 2 and 4 days after initial outreach (at 2 pm and 6:30 pm, respectively).

### Study Outcomes

The primary outcome was the percentage of patients who completed the first dose of the COVID-19 vaccine within 1 month of initial outreach. Secondary outcomes were the completion of the first dose within 2 months and completion of the vaccination series (2 doses of the mRNA-1273 [Moderna] or BNT162b2 [BioNTech-Pfizer] vaccine or 1 dose of the Ad.26.COV2.S [Janssen] vaccine) within 2 months of initial outreach. Additional outcomes included the percentage of patients with invalid cell phone numbers (wrong number or nontextable), no response to text messaging, the percentage of patients scheduled for the vaccine, text message responses (YES, INFO, NOT NOW, or DONE), and number of telephone calls made by the access center. Vaccine data were obtained from the EHR through automated data extraction that includes data at our own health system and queries from vaccine registries.

### Statistical Analysis

We estimated a 5% response rate to the telephone call–only group based on prior response rates to vaccine outreach at this health system. For the scheduling modality comparison, we compared each text message group (n = 10 000) with the telephone call group (n = 500), resulting in 2 comparisons (group 2 vs group 1; and group 3 vs group 1). Accounting for 2 pairwise comparisons with a *P* value threshold of .025 to maintain the overall .05 type I error level (Bonferroni correction, .05/2), we estimated more than 85% power to detect a difference of 4 percentage points (response rate of 9%) using the χ^2^ test of proportions, which was also based on preliminary analysis of prior health system text messaging efforts. We ended up with fewer participants than expected in the final analysis because we excluded patients who were found after study completion to have been vaccinated before randomization.

For the text messaging comparison, we allocated 5000 patients to each combined group (eg, standard messaging to the outbound call group and the inbound call group; clinician endorsement to the outbound call group and the inbound call group). We compared the clinician endorsement, scarcity framing, and endowment framing groups with the standard messaging group with 3 pairwise comparisons. Assuming a Bonferroni-corrected *P* value threshold of .02 (.05/3) and a baseline response rate of 8% for the standard text messaging, we estimated approximately 87% power to detect a difference of 2 percentage points (response rate of 10% for intervention groups) using the χ^2^ test of proportions. We did not anticipate interaction between scheduling workflow and messaging groups.

For the main intention-to-treat comparisons, we used the χ^2^ test of proportions to calculate difference and 95% CIs, along with *P* value. Demographics were obtained from the EHR through automated data extraction. Race and ethnicity were based on self-reported data in the EHR. Household income was estimated using the American Community Survey 2015-2019 5-Year Estimates data for median income by zip code of residence.

For the subgroup analyses, we developed a multivariable logistic model adjusting for sex (male or female), age (18-29, 30-39, 40-49, 50-64, or ≥65 years), race (Asian or Pacific Islander, Black, White, or other [includes American Indian, other, unknown, and refused]), ethnicity (Hispanic, not Hispanic, or other [includes unknown and refused]), household median income (<$35 000 or unknown, $35 000-$59 999, $60 000-$76 099, or ≥$76 100), insurance type (commercial, Medicaid, Medicare, or other), patient portal status (active or inactive), and patient’s prior visit date (≤2 years or >2 years). Interaction terms were added between each treatment group and each control variable and examined one heterogeneity covariate at a time, and likelihood ratio tests were conducted to examine goodness of fit for each model. On the basis of the estimated parameters in the fitted logistic models, we assessed treatment effect sizes of each intervention compared with the controls. The treatment effect sizes were defined as the difference between adjusted treatment effects of intervention and control groups, and results are presented in log odds ratios with 95% CIs. Analyses were performed in Stata, version 16.0 (StataCorp LLP)^20^ or Statsmodels, version 0.13.0 running on Python, version 3.9.7 (Python Software Foundation).^21,22^

## Results

### Participants

A total of 16 045 patients were included in the final analysis; the mean (SD) age was 36.9 (11.1) years; 9418 (58.7%) were women; 12 869 (80.2%) had commercial insurance and 2283 (14.2%) were insured by Medicaid; 4706 (29.3%) were Black, 967 (6.0%) were Hispanic or Latino, and 8345 (52.0%) were White (Table 1). A total of 19 554 patients were initially randomized; 3509 (17.9%) were later found to have completed at least 1 dose of the vaccine prior to study start (Figure). The proportion of patients who had completed at least 1 dose of the vaccine prior to study start was evenly distributed across study groups (eTable 1 in Supplement 2). In the EHR, 91.6% of patients (14 694 of 16 045) had an active email address recorded, and 81.4% (13 062 of 16 045) were active users of the patient portal. The intervention was conducted from April 29 through July 6, 2021, when the 2-month follow-up period was completed for all participants.

**Table 1.** Demographic Characteristics of Participants by Study Group

Characteristic	Participants, No. (%)									
	Outbound telephone call only (n=390)	Text message plus outbound telephone call				Text message plus inbound telephone call				Total (n=16 045)
		Standard messaging (n=1971)	Clinician endorsement (n=1985)	Scarcity (n=1977)	Endowment (n=1957)	Standard messaging (n=1918)	Clinician endorsement (n=1935)	Scarcity (n=1934)	Endowment (n=1978)	
Age, mean (SD), y	36.3 (11.6)	36.7 (10.9)	36.9 (10.6)	36.9 (11.2)	37.4 (11.6)	36.7 (11.0)	37.3 (11.2)	36.7 (11.3)	36.6 (10.9)	36.9 (11.1)
Sex										
Male	155 (39.7)	809 (41.1)	792 (39.9)	794 (40.2)	842 (43.0)	802 (41.8)	830 (42.9)	781 (40.4)	822 (41.6)	6627 (41.3)
Female	235 (60.3)	1162 (59.0)	1193 (60.1)	1183 (59.8)	1115 (57.0)	1116 (58.2)	1105 (57.1)	1153 (59.6)	1156 (50.4)	9418 (58.7)
Race										
Asian or Pacific Islander	26 (6.7)	143 (7.3)	161 (8.1)	139 (7.0)	157 (8.0)	152 (7.9)	153 (7.9)	159 (8.2)	150 (7.6)	1240 (7.7)
White	189 (48.5)	1040 (52.8)	1024 (51.6)	1023 (51.8)	1030 (52.6)	991 (51.7)	1017 (52.6)	1009 (52.2)	1022 (51.7)	8345 (52.0)
Black	127 (32.6)	568 (28.8)	597 (30.1)	598 (30.3)	575 (29.4)	563 (29.4)	550 (28.4)	553 (28.6)	575 (29.1)	4706 (29.3)
>1 Race	3 (0.8)	13 (0.7)	14 (0.7)	9 (0.5)	14 (0.7)	15 (0.8)	15 (0.8)	8 (0.4)	13 (0.7)	104 (0.7)
Other^a^	20 (5.1)	77 (3.9)	77 (3.9)	96 (4.9)	74 (3.8)	76 (4.0)	79 (4.1)	84 (4.3)	81 (4.1)	664 (4.1)
Unknown^b^	25 (6.4)	130 (6.5)	112 (5.6)	112 (5.7)	107 (5.5)	121 (6.3)	121 (6.3)	121 (6.3)	137 (6.9)	986 (6.1)
Ethnicity										
Hispanic or Latino	26 (6.7)	131 (6.7)	114 (5.7)	115 (5.8)	126 (6.4)	103 (5.4)	131 (6.8)	114 (5.9)	107 (5.4)	967 (6.0)
Not Hispanic or Latino	352 (90.3)	1785 (90.6)	1824 (91.9)	1829 (92.5)	1800 (92.0)	1781 (92.9)	1761 (91.0)	1779 (92.0)	1815 (91.8)	14 726 (91.8)
Unknown^b^	12 (3.1)	55 (2.8)	47 (2.4)	33 (1.7)	31 (1.6)	34 (1.8)	43 (2.2)	41 (2.1)	56 (2.8)	352 (2.2)
Patient portal status										
Active	308 (79.0)	1587 (80.5)	1618 (81.5)	1601 (81.0)	1613 (82.4)	1547 (80.7)	1582 (81.8)	1561 (80.7)	1645 (83.2)	13 062 (81.4)
Not active	82 (21.0)	384 (19.5)	367 (18.5)	376 (19.0)	344 (17.6)	371 (19.3)	353 (18.2)	373 (19.3)	333 (16.8)	2983 (18.6)
Insurance coverage type										
Commercial	308 (79.0)	1603 (81.3)	1558 (78.5)	1583 (80.1)	1565 (80.0)	1546 (80.6)	1575 (81.4)	1548 (80.0)	1583 (80.0)	12 869 (80.2)
Medicare	5 (1.3)	31 (1.6)	34 (1.7)	34 (1.7)	39 (2.0)	23 (1.2)	36 (1.9)	28 (1.5)	25 (1.3)	255 (1.6)
Medicaid	59 (15.1)	267 (13.6)	290 (14.6)	290 (14.7)	288 (14.7)	281 (14.7)	247 (12.8)	279 (14.4)	282 (14.3)	2283 (14.2)
Other^c^	2 (0.5)	11 (0.6)	17 (0.9)	17 (0.9)	11 (0.6)	11 (0.6)	22 (1.1)	17 (0.9)	23 (1.2)	131 (0.8)
Unknown	16 (4.1)	59 (3.0)	86 (4.3)	53 (2.7)	54 (2.8)	57 (3.0)	55 (2.8)	62 (3.2)	65 (3.3)	507 (3.2)
Household income, median (IQR), $^d^	54 438 (34 579-76 103)	57 735 (34 579-76 103)	53 255 (34 579-76 103)	52 001 (34 579-76 103)	57 126 (34 579-76 103)	57 126 (34 579-76 103)	57 735 (34 579-76 103)	57 126 (34 579-76 103)	57 735 (34 579-76 103)	57 126 (34 579-76 103)

^a^ Includes American Indian and other race or ethnicity.

^b^ Includes unknown and refused.

^c^ Includes no insurance and other.

^d^ American Community Survey (2015-2019) Median Household Income at zip code level in 2019 inflation-adjusted dollars. Data were missing for 25 participants.

**Figure.**
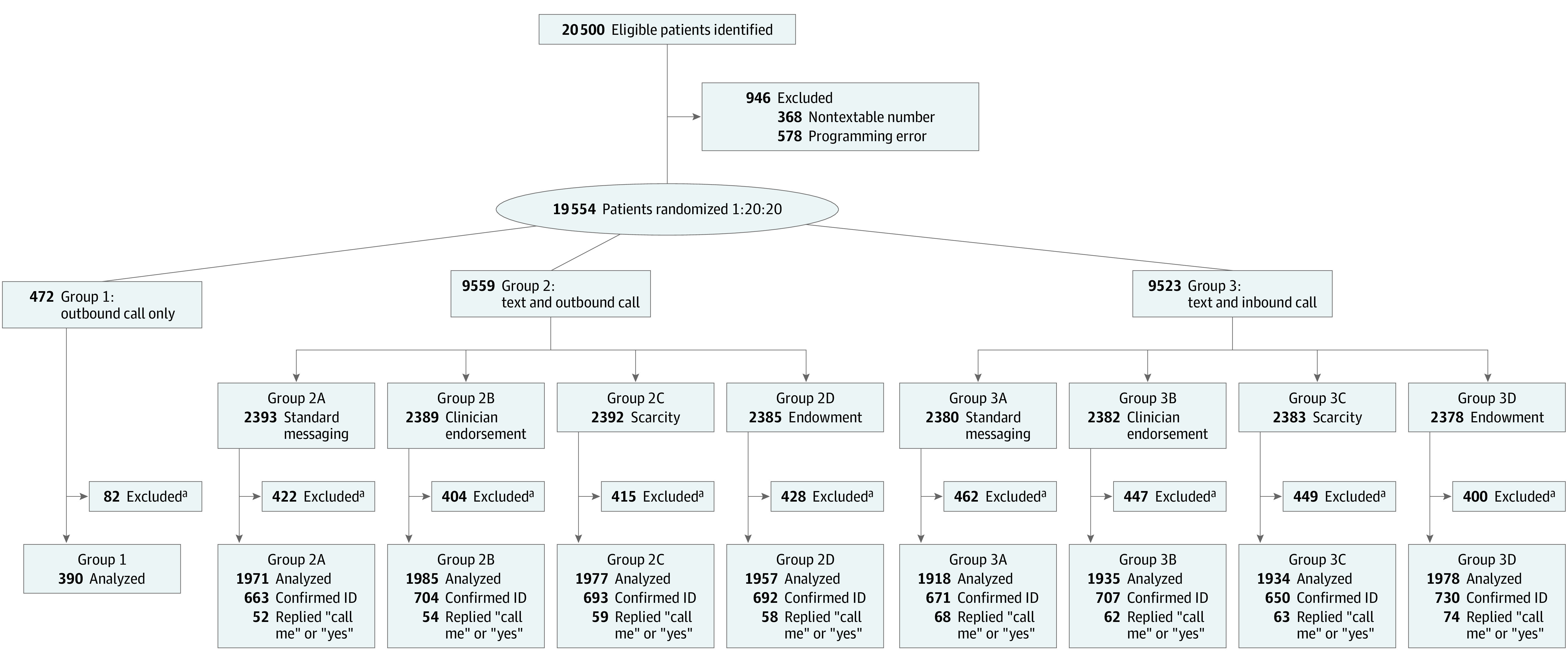
Consolidated Standards of Reporting Trials Flow Diagram ID indicates identification. ^a^Excluded for receiving 1 or more vaccine doses prior to outreach.

### Vaccine Completion

At 1 month, 14 of 390 patients (3.6%; 95% CI, 1.7%-5.4%) completed a vaccine dose in the outbound telephone only group. There was no significant increase in patient response among the text plus outbound call group (243 of 7890 patients [3.1%; 95% CI, 2.7%-3.5%]; absolute difference, −0.5% [95% CI, −2.4% to 1.4%; *P* = .57) or the inbound call group (253 of 7765 patients [3.3%; 95% CI, 2.9%-3.7%]; absolute difference, −0.3% [95% CI, −2.2% to 1.6%]; *P* = .72) (Table 2).

**Table 2.** Vaccine Completion in 1 and 2 Months by Scheduling Modality

Modality	No.	Dose completion at 1 mo			Dose completion at 2 mo			Series completion at 2 mo^a^		
		Completion, No. (%) [95% CI]	Difference, % (95% CI)	*P* value^b^	Completion, No. (%) [95% CI]	Difference, % (95% CI)	*P* value^b^	Completion, No. (%) [95% CI]	Difference, % (95% CI)	*P* value^b^
Outbound telephone call only	390	14 (3.6) [1.7 to 5.4]	NA	NA	16 (4.1) [2.1 to 6.1]	NA	NA	13 (3.3) [1.6 to 5.1]	NA	NA
Text plus outbound telephone call	7890	243 (3.1) [2.7 to 3.5]	−0.5 (−2.4 to 1.4)	.57	315 (4.0) [3.6 to 4.4]	−0.1 (−2.1 to 1.9)	.91	222 (2.8) [2.4 to 3.2]	−0.5 (−2.3 to 1.3)	.55
Text plus inbound telephone call	7765	253 (3.3) [2.9 to 3.7]	−0.3 (−2.2 to 1.6)	.72	342 (4.4) [3.9 to 4.9]	0.3 (−1.7 to 2.3)	.78	231 (3.0) [2.6 to 3.4]	−0.4 (−2.2 to 1.5)	.69

Abbreviation: NA, not applicable.

^a^ Completed 2 doses of the mRNA-1273 [Moderna] or BNT162b2 [BioNTech-Pfizer] vaccine or 1 dose of the Ad.26.COV2.S [Janssen] vaccine.

^b^ *P* < .025 was the threshold for statistical significance using the Bonferroni correction for multiple comparisons and a type I error rate of .05 (.05/2).

Among patients receiving text messaging, 118 of 3889 patients in the standard messaging group (3.0%; 95% CI, 2.5%-3.6%) completed 1 vaccine dose at 1 month. There was no significant increase in vaccine uptake among the clinician endorsement group (135 of 3920 patients [3.4%; 95% CI, 2.9%-4.0%]; absolute difference, 0.4% [95% CI, −0.4% to 1.2%]; *P* = .31), scarcity group (100 of 3911 patients [2.6%; 95% CI, 2.1%-3.1%]; absolute difference, −0.5% [95% CI, −1.2% to 0.3%]; *P* = .20), or endowment group (143 of 3935 patients [3.6%; 95% CI, 3.0%-4.2%]; absolute difference, 0.6% [95% CI, −0.2% to 1.4%]; *P* = .14) (Table 3).

**Table 3.** Vaccine Completion in 1 and 2 Months by Messaging Content

Messaging content	No.	Dose 1 completion at 1 mo			Dose 1 completion at 2 mo			Series completion at 2 mo^a^		
		Completion, No. (%) [95% CI]	Difference, % (95% CI)	*P* value^b^	Completion, No. (%) [95% CI]	Difference, % (95% CI)	*P* value^b^	Completion, No. (%) [95% CI]	Difference, % (95% CI)	*P* value^b^
Standard messaging	3889	118 (3.0) [2.5 to 3.6]	NA	NA	158 (4.1) [3.4 to 4.7]	NA	NA	104 (2.7) [2.2 to 3.2]	NA	NA
Clinician endorsement	3920	135 (3.4) [2.9 to 4.0]	0.4 (−0.4 to 1.2)	.31	177 (4.5) [3.9 to 5.2]	0.5 (−0.4 to 1.4)	.32	127 (3.2) [2.7 to 3.8]	0.6 (−0.2 to 1.3)	.14
Scarcity	3911	100 (2.6) [2.1 to 3.1]	−0.5 (−1.2 to 0.3)	.20	141 (3.6) [3.0 to 4.2]	−0.5 (−1.3 to 0.4)	.29	95 (2.4) [1.9 to 2.9]	−0.2 (−0.9 to 0.5)	.49
Endowment	3935	143 (3.6) [3.0 to 4.2]	0.6 (−0.2 to 1.4)	.14	181 (4.6) [3.9 to 5.3]	0.5 (−0.4 to 1.4)	.24	127 (3.2) [2.7 to 3.8]	0.6 (−0.2 to 1.3)	.15

Abbreviation: NA, not applicable.

^a^ Completed 2 doses of the mRNA-1273 [Moderna] or BNT162b2 [BioNTech-Pfizer] vaccine or 1 dose of the Ad.26.COV2.S [Janssen] vaccine.

^b^ *P* < .025 was the threshold for statistical significance using the Bonferroni correction for multiple comparisons and an overall type I error rate of .05 (.05/3).

At 1 month, 66.1% of the patients (337 of 510) who participated in the primary outcome completed their vaccination at our health system, with similar in-system completion rates by study group. The overall rate of 1 vaccine dose at 2 months was 4.2% (673 of 16 045) (Table 2). There were no significant differences in first dose completion or vaccine series completion at 2 months between scheduling or messaging groups (Table 2 and Table 3). Of the 673 patients who received at least 1 dose within 2 months, 466 (69.2%) completed their vaccine series during the 2-month time frame.

### Subgroup Analyses

We did not find meaningful differences in the scheduling group comparisons by subgroup characteristics, including sex, age, race and ethnicity, insurance type, timing of last visit, patient portal status, and income (eTable 2 in Supplement 2). There were also no meaningful differences by subgroup in the messaging groups (eTable 3 in Supplement 2). Although some of the isolated interaction effect sizes may appear significant, this finding is likely a result of multiple tests, and the overall likelihood ratio tests for overall fit were not significant (except for ethnicity in the messaging group test, which does not appear to be clinically relevant). In the messaging groups, Black patients had an overall higher response to all outreach (262 of 4706 [5.6%]) compared with White patients (162 of 8345 [1.9%]). In comparing responders with nonresponders, we found that patients who were Black, were older, had lower income, and had Medicaid were more likely to respond to overall outreach in the trial compared with other groups.

### Telephone Call and Text Message Results

A total of 15 655 patients were sent any text message outreach. Of those, 1043 patients (6.7%) indicated they were not the correct patient, 5118 (32.7%) did not reply to any text, and 426 (2.7%) did not reply to the offer after confirming their identity. Because patients were able to reply to the offer message more than once, eTable 4 in Supplement 2 displays the final adjudicated text outcome for each patient group; 3661 (23.4%) indicated they had completed vaccination (“DONE”), and an additional 81 (0.5%) replied to otherwise indicate that vaccination was complete.

The access center completed a total of 573 telephone calls to 459 patients across all study groups. Of the 273 patients in the outbound telephone call–only group who received a telephone call, 6 patients (2.2%) scheduled vaccination, 116 (42.5%) declined, and 150 (54.9%) were unreachable. Of the 178 patients in the text plus outbound call group who replied “YES” or “CALL ME,” 52 patients (29.2%) scheduled vaccination, 30 (16.9%) declined an appointment, and 85 (47.8%) were unreachable. Eight patients in the text plus inbound call group also received outbound calls. Two scheduled vaccination, 3 were already scheduled, 1 declined, and 2 were unreachable.

## Discussion

Our main finding was that text messaging scheduling outreach did not result in statistically detectable increases in vaccine uptake compared with outbound telephone calls. In addition, there was no statistically significant increase in vaccine uptake with behaviorally informed messaging compared with standard content. Across all forms of outreach, the overall response rate was 3.2% (510 of 16 045) at 1 month and 4.2% (673 of 16 045) at 2 months, which is lower than was anticipated when the trial was launched.

There are a few reasons that might explain the null findings. This trial took place from April to July 2021, when the vaccines were already available in Philadelphia. There were considerable efforts by public health agencies and other health systems to increase vaccine uptake. Our health system had also reached out to patients via email and the electronic patient portal, so the trial participants included those who were nonresponders and less likely to respond to another outreach attempt, or patients who did not have an email or patient portal account. The lower-than-anticipated response rate made it more difficult to detect differences in response to the different interventions. There is also the possibility that the privacy requirement for patients receiving text messaging to confirm identity may have added friction to the process and reduced the response rate. Owing to the public health imperative in increasing vaccine uptake, all patients in the trial received an active intervention of outreach, so we did not compare with a no intervention control group.

We found that the text messaging groups had similar rates of response to the outbound telephone call outreach group. Text messaging is low cost and allows more efficient deployment of limited access center staff by triaging patients who have interest in the vaccine. From an equity perspective, outbound telephone calls or text messaging are the only option for patients without email or internet access. For example, Black patients had an overall response rate of 5.6%, compared with 1.9% for White patients, and patients with Medicaid and those with lower incomes also had higher response rates. These groups were less likely to respond to earlier outreach from the health system that was based on email or patient portal messages, which may demonstrate an advantage of text messaging and telephone calls for certain groups.

Although the results were not statistically significant, the clinician endorsement and endowment groups had higher response rates than the other content. There is survey evidence that patients would trust their clinician’s recommendation regarding the COVID-19 vaccine,^16^ and identifying the primary care clinician’s name makes this explicit. Describing that the vaccine is reserved for the patient in the endowment message implies that the default is for the patient to receive the vaccine, and the patient may feel a sense of social obligation to receive the reserved vaccine.

A large trial of text messaging outreach by Dai et al^23^ showed that text messaging increased COVID-19 vaccine completion by 3.6 percentage points, but the baseline response rate to the invitation with no text messaging was much higher at 13.9%, because it was offered starting in January 2021. They also found a 1.1–percentage-point increase from “ownership” content that was similar to our endowment messaging. Another study by Santos et al^24^ that offered email outreach to health system employees for the COVID-19 vaccine in January 2021 showed about a 7% vaccine scheduling rate compared with 3% in the control group, but scheduling was measured over 3 days. These trials were conducted early in vaccine outreach when community demand was higher, while our trial was later in the rollout. They also compared the intervention group with a control group who did not receive outreach, while our trial had an active comparator group. A large trial in Rhode Island conducted at a similar time during vaccine rollout showed a null effect from behaviorally informed text messaging when compared with no outreach.^25^ A large trial by Milkman et al^10^ of text messaging before a primary care appointment to encourage influenza vaccination showed that endowment-framed messaging had the highest response rate, with a similar magnitude of effect to our messaging.

### Limitations

This study has some limitations. It was conducted at an academic health system with existing primary care patients, although the lookback interval was expansive at 5 years; however, most of these patients were seen in the last 2 years. Most patients included in this trial had already received vaccine-related outreach via email or patient portal, so they were particularly difficult to reach. Although we were able to use registry data to track vaccines outside of our health system, we likely missed some vaccine outcomes outside of this network. One-fourth of the text messaging responses stated that they already completed the vaccine; this would have biased the findings toward the null. Also, the number of patients in the final analysis was less than our anticipated sample size in the power calculations owing to exclusions.

## Conclusions

In this randomized clinical trial embedded in health system operations, we found that text messaging is a feasible modality for communication and scheduling in large-scale population health outreach initiatives. However, the response rate was low and we did not find significant differences by scheduling modality or messaging content, although there was no control group of individuals who did not receive an intervention. Additional interventions that address vaccine hesitancy, encourage uptake, and make it easier to receive the vaccination are needed.

## Notes

Supplement 1.
Trial Protocol

Supplement 2. **eTable 1.** Vaccine Received Prior to Outreach **eTable 2.** Dose 1 Completion in One Month by Subgroup (Scheduling Modality) **eTable 3.** Dose 1 Completion in One Month by Subgroup (Messaging Content) **eTable 4.** Final Text Response

Supplement 3.
Data Sharing Statement
