# Supplementary material for: Effect of Text Messaging and Behavioral Interventions on COVID-19 Vaccination Uptake: A Randomized Clinical Trial
Source: JAMA Netw Open. 2022 Jun 13;5(6):e2216649. doi: 10.1001/jamanetworkopen.2022.16649 (PMC9194662; doi:10.1001/jamanetworkopen.2022.16649)
Supplement: Supplement 1. — Trial Protocol [file jamanetwopen-e2216649-s001.pdf]

Supplement

Pragmatic Trial of COVID Vaccine Text Outreach Interventions

This supplement provides additional information about the work. It contains the following items:

Initial Protocol ………2

Final Protocol ………11

Summary of protocol changes ………………………………………………………………………………………20

Original statistical analysis plan ……………………………………………………………………………………22

Final statistical analysis plan …………………………………………………………………………………………23

Summary of statistical analysis plan changes ……………………………………………………………………24

Appendix A: Participant & Provider Messaging ……………………………………………………………………25

Pragmatic Trial of COVID Vaccine Text Outreach Interventions

Brief Description

This project aims to evaluate different approaches to increase COVID-19 vaccine uptake among at-risk patients at Penn Medicine in an equitable and systematic manner through a centralized outreach program. We will evaluate different text-based scheduling and messaging approaches informed by behavioral science to increase uptake among eligible patients, including the use of clinician endorsement, opt-out framing, and scarcity.

Abstract

COVID-19 is a significant cause of global morbidity and mortality. The COVID-19 vaccine can effectively reduce significant morbidity and eliminate mortality in vaccinated populations, but the pandemic has exacerbated existing racial and socioeconomic disparities in access to and uptake of the COVID-19 vaccine. In this project, we will evaluate different ways to reach out to at-risk patients to encourage them to participate in vaccination, comparing different scheduling workflows and messaging approaches informed by behavioral science with the goal of increasing COVID-19 vaccine uptake.

Data Management

Information about study subjects will be kept confidential and managed according to the requirements of the Health Insurance Portability and Accountability Act of 1996 (HIPAA). Source documents are maintained in PennChart. No source documents will be printed or maintained in paper form at the study site. Data from PennChart will be recorded in Penn Medicine's REDCap system. The investigator and study team will have access to PHI within PennChart and REDCap. We will label all PHI within REDCap as identifiable information so that de-identified exports are possible. All reports that include identifiable information will be stored on the Innovation Center secure drive, maintained behind the UPHS firewall. Direct identifiers will be maintained on RedCap until manuscript publication in case additional chart review is needed for confirmation of results. Once data analysis and manuscripts have been published, direct identifiers will be deleted from RedCap and the de-identified database will be stored on the Innovation Center secure drive.

Objectives

We will evaluate a centralized approach to increasing COVID through direct outreach to eligible patients via text messaging. This pragmatic randomized controlled trial has the following aims:

Aim 1: To conduct systematic large scale text message outreach for vaccine delivery among a defined group of Penn Medicine in a way that advances equity.
Aim 2: To offer different scheduling workflows through text messaging that might increase uptake and reduce disparities.
Aim 3: To compare the equity and efficiency of different messages informed by behavioral science, including PCP endorsement, opt-out framing, and scarcity.

Primary outcome variable(s)

The primary outcome will be the percentage of patients who complete the first dose of the COVID vaccine within 1 month of initial outreach.

Secondary outcome variable(s)

Secondary outcomes will be the completion of the first dose within 2 months, and completion of the vaccination process within 2 months of initial outreach. We will also examine the percentage of patients that are scheduled, text message responses (YES, INFO, NOT NOW, DONE), and number of phone calls made by the access center. We will compare response rates by age, race/ethnicity, sex, insurance type, and income (by zip code).

Background

The national roll-out of the COVID-19 vaccine has already revealed reduced uptake among Black patients due to barriers in the technical and logistical burden of vaccination, and overall hesitancy about receiving the vaccine. Those barriers are not distributed uniformly. Penn Medicine has played an integral role in delivering the COVID-19 vaccine to our community, but it will be challenging to ensure our approach does not exacerbate existing racial and socioeconomic disparities in the pandemic and, instead, helps overcome them.

The COVID vaccine workgroup at Penn Medicine (including the Office of CMIO, CPUP, PMMG, Access Center, Marketing, and CHCI) has identified approximately 19,000 patients with a cell phone number who are not enrolled in MyPennMedicine (MPM) and do not have an email address in the EHR and approximately 95,000 patients who have been sent an MPM message or email and have not yet scheduled an appointment. The team has already developed and implemented a protocol to send text messages (using the Way to Health platform) to offer the vaccine and help schedule through collaboration with the Access Center. Through this program, we propose to prospectively randomize scheduling workflow and messaging to identify the most effective and equitable approaches to increasing vaccine uptake. What we propose will simultaneously [1] identify the interventions with the highest response rate that can inform Penn Medicine efforts locally and [2] conduct a pragmatic trial with a rigorous analysis plan that we can broadly apply to other contexts and share with other health systems.

Study Design

Design

This is a pragmatic randomized controlled trial with a factorial design. 21,000 patients will be included and randomized to 3 study arms related to scheduling workflow in a 1:10:10 ratio to include 1,000 patients in the usual care arm (phone call only) and 10,000 patients in each text messaging arm, stratified by whether they have already received an email or MPM as follows: 1) Phone Call (usual care), 2) Opt-In (call back), 3) Preference (preference + call back). Among the 20,000 patients receiving text messaging (Arms 2&3), patients will be additionally randomized in a factorial design to four different types of messaging content informed by principles of behavioral science in a 1:1:1:1 ratio (5,000 in each arm), stratified by whether they have already received an email or MPM inviting them to schedule for vaccination: A) Standard Messaging, B) Clinician Endorsement, C) Scarcity, and D) Opt-Out Framing.

Study duration

We anticipate 2 weeks to set up the text messaging platform, and to identify and randomize eligible patients. Phone calls and text messaging will begin at the same time, and initial outreach and all follow-up messaging will be completed within 35 days from initial outreach.

Resources necessary for human research protection

Dr. Shivan Mehta is the PI of this study. He is an assistant professor of medicine at the Perelman School of Medicine, University of Pennsylvania and Associate Chief Innovation Officer at Penn Medicine. All members of the research team have completed CITI human subjects research training. Standard Operating Procedure documents for the project will be accessible to all members of the research team, which will keep research staff informed about the protocol and their related duties. There are adequate facilities to conduct the research.

Characteristics of the Study Population

Target population

This program will take place across patients seen at Penn Medicine practices who reside in the city of Philadelphia.

Subjects enrolled by Penn Researchers

21,000

Subjects enrolled by Collaborating Researchers

0

Subject Recruitment

21,000 patients will be identified through automated data extraction from the electronic health record (EHR). All eligible patients will receive vaccine outreach via phone call or text message. Patients will be stratified by whether they have previously received an email or MPM message to schedule their vaccination. In order to minimize the outgoing phone call burden on the access center, we will randomize 1,000 patients in the phone call arm (Arm 1). The remaining 20,000 patients will be randomized to receive text message based outreach.

Accrual

We will conduct the study in close partnership and with approval from the COVID vaccine workgroup for messaging and outreach methods. As a pragmatic trial, we are randomly selecting 21,000 eligible patients followed by Penn Medicine per the current city of Philadelphia eligibility guidelines for vaccination.

Key inclusion criteria:
Patients who reside in Philadelphia who have had at least 1 visit in the past 3 years at a Penn Medicine clinical practice and who are either age >=65 or, 16-64 with a chronic medical condition (Diabetes, Chronic Kidney Disease, Sickle Cell Disease, Chronic Obstructive Pulmonary Disorder (not including

asthma), Heart Conditions, Obesity (>30 BMI), Severe Obesity, Smoker, HIV, Pregnancy, or Down Syndrome).

Key exclusion criteria:
Patients will be excluded if they have completed any dose of the vaccine at Penn Medicine or are currently scheduled.

Procedures

We request a waiver of informed consent as this is low-risk, supported by and embedded in clinical operations, and we could not practicably obtain consent. Through automated data extraction, we will identify eligible patients, along with the name of their primary care provider or the last clinician that saw them in a visit. All patients will receive outreach about scheduling an appointment to receive the COVID-19 vaccine.

<u>Randomization:</u>
21,000 patients will be stratified by whether they have previously received an email or MPM message inviting them to schedule for vaccination or not, then randomized to 3 study arms related to scheduling workflow in a 1:10:10 ratio (1,000 usual care, 10,000 in intervention arms) to receive: 1) Phone Call (usual care), 2) Opt-In (call back), 3) Preference (preference + call back).

- *Arm 1: Phone Call (usual care):* 1,000 patients will be randomized to Arm 1, Phone Call (usual care) and will receive a phone call to schedule their appointment from an Access Center representative. Access Center representatives will make up to 3 attempts to schedule an appointment with the patient. Patients randomized to this arm will not receive any text messaging.

The remaining 20,000 patients will be randomized to study arms that will receive one of 3 scheduling workflows in a 1:1:1 ratio (10,000 in each arm):

- *Arm 2: Opt-In (call back):* Messaging will include a prompt to agree to scheduling: "If you would like us to call you to schedule your appointment please reply, YES." The access center will call these patients back to schedule, calling up to 3 times.
- *Arm 3: Preference (Preference + call back):* Messaging will include a prompt to agree to scheduling, which will be following by a prompt to identify preferred days and time slots for the vaccine (M-F, AM/PM). The access center will call these patients back to schedule, calling up to 3 times.

Among Arms 2-3, patients will be additionally randomized to different messaging content informed by principles of behavioral science in a 1:1:1:1 ratio (5,000 in each arm), stratified by whether they have already received an email or MPM message to schedule their vaccination:

- *Arm A: Standard messaging:* The message describes that the patient is eligible for the COVID vaccine. ("Our records show you are eligible for your COVID-19 vaccine at Penn Medicine.")
- *Arm B: Clinician endorsement*: The message will describe an endorsement from the provider to get the vaccination. ("Dr. XXXX recommends that you receive the vaccination.")

- *Arm C: Scarcity*: The message will highlight the limited availability and the elevated priority for the patient to receive the vaccine at Penn Medicine. ("You have been selected to receive from the limited supply of COVID-19 vaccine at Penn Medicine.")
- *Arm D: Opt-out framing:* This will highlight that a vaccine is reserved for the patient, implying that they need to opt-out ("We have reserved a COVID-19 vaccine appointment for you at Penn Medicine.").

All study arms will be further randomized into 4 smaller batches of 1,250 patients each (250 in the phone call only arm). All patients assigned to batch 1 will receive messages on the same day, per their study arm assignment. A total of 5,000 initial outreach text messages and 250 phone calls will be sent each week for 4 weeks until all 21,000 patients have been contacted. Batched messaging will help evenly spread the call volume for the Access Center and help balance scheduling capacity with vaccine supply.

<u>Text Messaging:</u>
All eligible patients randomized to the text message arms will receive text messaging about the COVID vaccine from Penn Medicine. The first message will describe that it is from Penn Medicine, ask the patient to confirm their name, and allow for patients to opt-out of further outreach. If the patient confirms, the 2nd text message will describe that patients are eligible for the COVID 19 vaccine ("Our records show you are eligible for your COVID-19 vaccine at Penn Medicine."). This will be followed by a behaviorally-informed messaging prompt ("YES"), followed by the mechanism through which the patient can get scheduled for the vaccine. At the end of the message, there will also be a prompt for more information ("INFO"), to defer the vaccine for now ("NOT NOW"), or to indicate vaccination has already been completed ("DONE").

If no reply is received and the patient has not opted out of messaging or deferred the vaccine ("NOT NOW"), they will receive a reminder text consisting of the same scheduling flow and messaging content of their original message, 4 days after initial outreach.

Arms 2 & 3 require patients to opt-in to receive a call back from Access Center representatives. This information will be captured in the Way to Health platform and will be securely transmitted in a report to the Access Center daily.

If a patient in Arm 3 responds "YES" to the initial invitation text but does not respond to either of the preference prompts, they will still receive a scheduling phone call from the Access Center offering an appointment.

Questions that are submitted by patients via text will be manually reviewed and responded to or routed as appropriate.

Analysis Plan

The primary outcome will be the percentage of patients who complete the first dose of the COVID vaccine within 1 month of initial outreach. Secondary outcomes will be the completion of the first dose within 2 months, and completion of the vaccination process within 2 months of initial outreach. We will also examine the percentage of patients that are scheduled, text message responses (YES, INFO, NOT NOW, DONE), and number of phone calls made by the access center. We will compare response rates by

MPM/email status, age, race/ethnicity, sex, insurance, and income (by zip code).

We estimate a 5% response rate to the phone call only. In order to minimize the outgoing phone call burden on the access center, we will include 1,000 patients in the outbound phone call arm (Arm 1). For the scheduling flow comparison, we will allocate 10,000 patients in each text message arm (Arms 2-3). We will compare each text message arm to the phone call arm, resulting in 2 comparisons (arm 2 vs arm 1, arm 3 vs arm 1). Accounting for 2 pairwise comparisons with a P-value threshold of .025 (Bonferroni correction; .05/2), we estimate 91% power to detect a difference of 3 percentage points (response rate of 8%) using the chi-squared test of proportions [STATA: power twoprop .05 .08, n1(1000) n2(10000) a(.025)].

For the messaging comparison, we will allocate 5,000 patients in each arm. We will compare arms B-D to Arm A with 3 pairwise comparisons. Assuming a P-value threshold of .017 (.05/3) and a baseline response rate of 8% for text messaging, we estimate approximately 87% power to detect a difference of 2 percentage points (response rate of 10% for intervention arms) using the chi-squared test of proportions. [STATA: power twoprop .08 .10, n1(5000) n2 (5000) a(.017)]. We do not anticipate interaction between scheduling workflow and messaging arms.

Subject Confidentiality

Information about study subjects will be kept confidential and managed according to the requirements of the Health Insurance Portability and Accountability Act of 1996 (HIPAA). All PHI will be maintained on UPHS servers. Source documents are maintained in PennChart. No source documents will be printed or maintained in paper form at the study site. Data from PennChart will be recorded in Penn Medicine's REDCap system. The investigator and study team will have access to PHI within PennChart and REDCap. We will label all PHI within REDCap as identifiable information so that de-identified exports are possible. All reports that include identifiable information will be stored on the Innovation Center secure drive, maintained behind the UPHS firewall. Direct identifiers will be maintained on RedCap until manuscript publication in case additional chart review is needed for confirmation of results. Once data analysis and manuscripts have been published, direct identifiers will be deleted from RedCap and the de-identified database will be stored on the Innovation Center secure drive.

Sensitive Research Information

This Research does not involve collection of sensitive information about the subjects that should be excluded from the electronic medical record.

Subject Privacy

21,000 patients will be identified through automated data extraction from the electronic health record (EHR). The research team will have no direct interaction with study participants. Text messaging will be conducted via the Way to Health platform. The initial text message will confirm identity prior to proceeding with additional text messaging for the intervention.

Data Disclosure
Patients who agree to scheduling an appointment for COVID-19 vaccination will receive up to 3 phone calls from Penn Medicine Access Center representatives in an attempt to schedule them for their appointment. As such, patients will be identified in EPIC via a scheduling queue for Access Center representative call queue purposes.

Consent

1. Consent Process

Overview

Waiver of consent for this study is requested. Please see below.

Children and Adolescents

Not applicable.

Adult Subjects Not Competent to Give Consent

Waiver of consent is being requested.

2. Waiver of Consent

Waiver or Alteration of Informed Consent*

Waiver or alteration of required elements of consent.

Minimal Risk*

This study involves no more than minimal risk to subjects. Outreach for COVID-19 vaccination is an operational imperative and this trial is embedded in routine clinical operations at Penn Medicine. All participants will receive messaging indicating they are eligible to receive the vaccine as well as a prompt for scheduling. The only research related activity is the randomization of patients to different types of scheduling and messaging strategies that will help inform best practices for future outreach to other patient populations. Participants must still consent to receive the vaccine at the time of vaccination, as is usual care.

Impact on Subject Rights and Welfare*

Subjects rights and welfare will not be adversely affected by the waiver of authorization and consent. All subjects will have the opportunity to voluntarily receive or not receive the COVID-19 vaccination. This trial aims to identify outreach approaches that might increase uptake.

Waiver Essential to Research*
We believe that we would not be able to practically conduct the research without waiver of consent. If we had to obtain either written or verbal consent ahead of time, it would substantially limit our study population and it may alter their participation in the intervention. Thus, we would only learn about the response rate for patients who we were able to speak to for consent. This would limit the generalizability to practice. We believe that subsequent completion of vaccination or deferral of participation in vaccination is still completely voluntary, and participants must provide consent at the time of vaccination. Obtaining waiver of consent would allow us to avoid the potential selection/volunteer bias for inclusion of patients particularly interested in vaccination that can occur

when consent is required. Since our main objective is to understand the potential influence varying outreach strategies on participant behavior, we believe that obtaining consent would not be practicable. Additionally, we have received waiver of consent for similar studies related to population health outreach in the past.

Additional Information to Subjects
Participants who are vaccinated will be provided with appropriate information about possible side-effects and follow-up as per current vaccine guidelines and Penn Medicine standards of care. Participants will be offered their 2nd dose appointment as appropriate per the type of vaccine given at the first appointment.

Written Statement of Research*
We do not believe it would be reasonable to inform patients of their participation in this behavioral outreach study upon completion, as conveying the intent of the research (that it is not a vaccine trial) to a lay population would likely lead to unnecessary confusion. Calling all patients would be infeasible, and sending a text message would not be a sufficient platform to explain the nuances of the study. Text messaging might also crowd out other more important messaging from Penn Medicine about health promotion. As this is a behavior observation study and all participants will receive outreach, we feel the risk of harm from informing participants is greater than not informing them.

Risk / Benefit

Potential Study Risks
The risks associated with this study are no more than minimal. There is the potential risk of breach of confidentiality. We will minimize this risk by using de-identified information whenever possible and by maintaining all identifiable information on a secure drive and/or in a HIPAA-compliant system (e.g. REDCap) as is already being done through routine clinical operations. There is also the risk of physical discomfort from the vaccine. We will minimize this risk by communicating the potential discomforts at the time of the vaccination and by offering routine monitoring, as is the current standard practice for COVID-19 vaccinated patients at Penn Medicine.

Potential Study Benefits
If a participant completes their COVID-19 vaccination, they will potentially benefit by dramatically reducing the likelihood of infection by COVID-19 causing severe complications, hospitalization or death. Additionally, their vaccination will potentially benefit the population by increasing the overall rate of immunity and slowing disease spread among those unable to receive the vaccine. Information learned from this study may additionally benefit society through a better understanding of how to effectively increase overall uptake of the COVID-19 vaccination, which could inform future vaccination campaigns or be applied to other population-wide contexts.

Alternatives to Participation (optional)
Participants may choose to opt-out of receiving text messaging and/or decline scheduling their vaccination.

Data and Safety Monitoring
Safety will be overseen by the PI and the study team. In the case of possible events, the PI or designee will review the study charts to evaluate events at each subject interaction to ensure the grade, relationship to the study procedure, expectedness, and the course of action for each subject is documented.

Risk / Benefit Assessment
The risks associated with this study are no more than minimal. Better knowledge of how to increase COVID-19 vaccine uptake could potentially address a major source of disparities among our patient population. Better vaccine uptake could potentially reduce the already increased disease burden on our most at-risk patients. For these reasons and those outlined in the above benefits section, the investigator believes that the risks of participating in the study are outweighed by the potential benefits of participating in the study.

Final Protocol

****New changes from initial protocol notated in bold, parts removed from initial protocol notated in strikethrough**

Brief Description

This project aims to evaluate different approaches to increase COVID-19 vaccine uptake among at-risk patients at Penn Medicine in an equitable and systematic manner through a centralized outreach program. We will evaluate different text-based scheduling and messaging approaches informed by behavioral science to increase uptake among eligible patients, including the use of clinician endorsement, opt-out framing, and scarcity.

Abstract

COVID-19 is a significant cause of global morbidity and mortality. The COVID-19 vaccine can effectively reduce significant morbidity and eliminate mortality in vaccinated populations, but the pandemic has exacerbated existing racial and socioeconomic disparities in access to and uptake of the COVID-19 vaccine. In this project, we will evaluate different ways to reach out to at-risk patients to encourage them to participate in vaccination, comparing different scheduling workflows and messaging approaches informed by behavioral science with the goal of increasing COVID-19 vaccine uptake.

Data Management

Information about study subjects will be kept confidential and managed according to the requirements of the Health Insurance Portability and Accountability Act of 1996 (HIPAA). Source documents are maintained in PennChart. No source documents will be printed or maintained in paper form at the study site. Data from PennChart will be recorded in Penn Medicine's REDCap system. The investigator and study team will have access to PHI within PennChart and REDCap. We will label all PHI within REDCap as identifiable information so that de-identified exports are possible. All reports that include identifiable information will be stored on the Innovation Center secure drive, maintained behind the UPHS firewall. Direct identifiers will be maintained on RedCap until manuscript publication in case additional chart review is needed for confirmation of results. Once data analysis and manuscripts have been published, direct identifiers will be deleted from RedCap and the de-identified database will be stored on the Innovation Center secure drive.

Objectives

We will evaluate a centralized approach to increasing COVID through direct outreach to eligible patients via text messaging. This pragmatic randomized controlled trial has the following aims:

<u>Aim 1</u>: To conduct systematic large scale text message outreach for vaccine delivery among a defined group of Penn Medicine in a way that advances equity.
<u>Aim 2</u>: To offer different scheduling workflows through text messaging that might increase uptake and reduce disparities.
<u>Aim 3</u>: To compare the equity and efficiency of different messages informed by behavioral science, including PCP endorsement, opt-out framing, and scarcity.

Primary outcome variable(s)

The primary outcome will be the percentage of patients who complete the first dose of the COVID vaccine within 1 month of initial outreach.

Secondary outcome variable(s)

Secondary outcomes will be the completion of the first dose within 2 months, and completion of the vaccination process within 2 months of initial outreach. We will also examine the percentage of patients that are scheduled, text message responses (YES, INFO, NOT NOW, DONE), and number of phone calls made by the access center. We will compare response rates by age, race/ethnicity, sex, insurance type, and income (by zip code).

Background

The national roll-out of the COVID-19 vaccine has already revealed reduced uptake among Black patients due to barriers in the technical and logistical burden of vaccination, and overall hesitancy about receiving the vaccine. Those barriers are not distributed uniformly. Penn Medicine has played an integral role in delivering the COVID-19 vaccine to our community, but it will be challenging to ensure our approach does not exacerbate existing racial and socioeconomic disparities in the pandemic and, instead, helps overcome them.

The COVID vaccine workgroup at Penn Medicine (including the Office of CMIO, CPUP, PMMG, Access Center, Marketing, and CHCI) has identified approximately 19,000 patients with a cell phone number who are not enrolled in MyPennMedicine (MPM) and do not have an email address in the EHR and approximately 95,000 patients who have been sent an MPM message or email and have not yet scheduled an appointment. The team has already developed and implemented a protocol to send text messages (using the Way to Health platform) to offer the vaccine and help schedule through collaboration with the Access Center. Through this program, we propose to prospectively randomize scheduling workflow and messaging to identify the most effective and equitable approaches to increasing vaccine uptake. What we propose will simultaneously [1] identify the interventions with the highest response rate that can inform Penn Medicine efforts locally and [2] conduct a pragmatic trial with a rigorous analysis plan that we can broadly apply to other contexts and share with other health systems.

Study Design

Design

This is a pragmatic randomized controlled trial with a factorial design. ~~21,000~~ **20,500** patients will be included and randomized to 3 study arms related to scheduling workflow in a ~~1:10:10~~ **1:20:20** ratio to include ~~1,000~~ **500** patients in the usual care arm (phone call only) and 10,000 patients in each text messaging arm~~, stratified by whether they have already received an email or MPM~~ as follows: 1) Phone Call (usual care), 2) Opt-In (call back), 3) ~~Preference~~ **Opt-In** (~~preference~~ inbound ~~+ call back~~). Among the 20,000 patients receiving text messaging (Arms 2&3), patients will be additionally randomized in a factorial design to four different types of messaging content informed by principles of behavioral science in a 1:1:1:1 ratio (5,000 in each arm~~), stratified by whether they have already received an email or MPM inviting them to schedule for vaccination~~: A) Standard Messaging, B) Clinician Endorsement, C) Scarcity, and D) Opt-Out Framing.

Study duration

We anticipate 2 weeks to set up the text messaging platform, and to identify and randomize eligible patients. Phone calls and text messaging will begin at the same time, and initial outreach and all follow-up messaging will be completed within 35 days from initial outreach.

Resources necessary for human research protection

Dr. Shivan Mehta is the PI of this study. He is an assistant professor of medicine at the Perelman School of Medicine, University of Pennsylvania and Associate Chief Innovation Officer at Penn Medicine. All members of the research team have completed CITI human subjects research training. Standard Operating Procedure documents for the project will be accessible to all members of the research team, which will keep research staff informed about the protocol and their related duties. There are adequate facilities to conduct the research.

Characteristics of the Study Population

Target population

This program will take place across patients seen at Penn Medicine practices who reside in the city of Philadelphia.

Subjects enrolled by Penn Researchers

~~21,000~~ **20,500**

Subjects enrolled by Collaborating Researchers

0

Subject Recruitment
~~21,000~~ **20,500** patients will be identified through automated data extraction from the electronic health record (EHR). All eligible patients will receive vaccine outreach via phone call or text message. ~~Patients will be stratified by whether they have previously received an email or MPM message to schedule their vaccination.~~ In order to minimize the outgoing phone call burden on the access center, we will randomize ~~1,000~~ **500** patients in the phone call arm (Arm 1). The remaining 20,000 patients will be randomized to receive text message based outreach.

Accrual

We will conduct the study in close partnership and with approval from the COVID vaccine workgroup for messaging and outreach methods. As a pragmatic trial, we are randomly selecting ~~21,000~~ **20,500** eligible patients followed by Penn Medicine per the current city of Philadelphia eligibility guidelines for vaccination.

Key inclusion criteria:
Patients **aged 18+** who reside in Philadelphia who have had at least 1 visit in the past ~~3~~ 5 years, **or a**

future scheduled visit within the next 3 months, ~~at~~ **with** a Penn Medicine **primary care provider on file.** ~~clinical practice and who are either age >=65 or, 16-64 with a chronic medical condition (Diabetes, Chronic Kidney Disease, Sickle Cell Disease, Chronic Obstructive Pulmonary Disorder (not including asthma), Heart Conditions, Obesity (>30 BMI), Severe Obesity, Smoker, HIV, Pregnancy, or Down Syndrome).~~

Key exclusion criteria:
Patients will be excluded if they have completed any dose of the vaccine at Penn Medicine**,** ~~or~~ are currently scheduled**, have externally documented vaccination records, or have previously received text-message based vaccine outreach**.

Procedures

We request a waiver of informed consent as this is low-risk, supported by and embedded in clinical operations, and we could not practicably obtain consent. Through automated data extraction, we will identify eligible patients, along with the name of their primary care provider or the last clinician that saw them in a visit. All patients will receive outreach about scheduling an appointment to receive the COVID-19 vaccine.

Randomization:
~~21,000~~ **20,000** patients will be ~~stratified by whether they have previously received an email or MPM message inviting them to schedule for vaccination or not, then~~ randomized to 3 study arms related to scheduling workflow in a ~~1:10:10~~ **1:20:20** ratio (~~1,000~~ **500** usual care, 10,000 in intervention arms) to receive: 1) Phone Call (usual care), 2) Opt-In (call back), 3) ~~Preference~~ Opt-In(~~preference + call back~~ Inbound).

- *Arm 1: Phone Call (usual care):* ~~1,000~~ **500** patients will be randomized to Arm 1, Phone Call (usual care) and will receive a phone call to schedule their appointment from an Access Center representative. Access Center representatives will make up to 3 attempts to schedule an appointment with the patient. Patients randomized to this arm will not receive any text messaging.

The remaining 20,000 patients will be randomized to study arms that will receive one of ~~3~~ **2** scheduling workflows in a 1:1:1 ratio (10,000 in each arm):

- *Arm 2: Opt-In (call back):* Messaging will include a prompt to agree to scheduling: "If you would like us to call you to schedule your appointment please reply, YES." The access center will call these patients back to schedule, calling up to 3 times.
- *Arm 3: ~~Preference (Preference + call back)~~ Opt-in (Inbound):* Messaging will include a prompt to agree to scheduling, which will be following by a prompt to ~~identify preferred days and time slots for the vaccine (M-F, AM/PM). The access center will call these patients back to schedule, calling up to 3 times~~ **call the Penn Medicine COVID-19 Vaccine Schedule Hotline**.

Among Arms 2-3, patients will be additionally randomized to different messaging content informed by principles of behavioral science in a 1:1:1:1 ratio (~~5,000~~ **2,500** in each arm)~~, stratified by whether they have already received an email or MPM message to schedule their vaccination~~:

- *Arm A: Standard messaging:* The message describes that the patient is eligible for the COVID vaccine. ("Our records show you are eligible for your COVID-19 vaccine at Penn Medicine.")
- *Arm B: Clinician endorsement*: The message will describe an endorsement from the provider to get the vaccination. ("Dr. XXXX recommends that you receive the vaccination.")
- *Arm C: Scarcity*: The message will highlight the limited availability and the elevated priority for the patient to receive the vaccine at Penn Medicine. ("You have been selected to receive from the limited supply of COVID-19 vaccine at Penn Medicine.")
- *Arm D: Opt-out framing:* This will highlight that a vaccine is reserved for the patient, implying that they need to opt-out ("We have reserved a COVID-19 vaccine appointment for you at Penn Medicine.").

All study arms will be further randomized into ~~4~~ **5** smaller batches of ~~1,250~~ **500** patients each (~~250~~ **100** in the phone call only arm). All patients assigned to batch 1 will receive messages on the same day, per their study arm assignment. A total of ~~5~~**4**,000 initial outreach text messages and ~~250~~ **100** phone calls will be sent each ~~week~~ ~~for 4 weeks~~ **day for 5 days** until all ~~21,000~~ **20,500** patients have been contacted. Batched messaging will help evenly spread the call volume for the Access Center and help balance scheduling capacity with vaccine supply.

<u>Text Messaging:</u>
All eligible patients randomized to the text message arms will receive text messaging about the COVID vaccine from Penn Medicine. The first message will describe that it is from Penn Medicine, ask the patient to confirm their name, and allow for patients to opt-out of further outreach. If the patient confirms, the 2nd text message will describe that patients are eligible for the COVID 19 vaccine ("Our records show you are eligible for your COVID-19 vaccine at Penn Medicine."). This will be followed by a behaviorally-informed messaging prompt ("YES"), followed by the mechanism through which the patient can get scheduled for the vaccine. At the end of the message, there will also be a prompt for more information ("INFO"), to defer the vaccine for now ("NOT NOW"), or to indicate vaccination has already been completed ("DONE").

If no reply is received and the patient has not opted out of messaging or deferred the vaccine ("NOT NOW"), they will receive **up to 2** ~~a~~ reminder text**s** ~~consisting of the same scheduling flow and messaging content of their original message,~~ **2 and** 4 days after initial outreach.

Arms 2 ~~& 3~~ **will** require patients to opt-in to receive a call back from Access Center representatives. **Arm 3 will require patients to opt-in to receive the vaccine scheduling hotline number and call to schedule themselves.** This information will be captured in the Way to Health platform and will be securely transmitted in a report to the Access Center daily.

~~If a patient in Arm 3 responds "YES" to the initial invitation text but does not respond to either of the preference prompts, they will still receive a scheduling phone call from the Access Center offering an appointment.~~

Questions that are submitted by patients via text will be manually reviewed and responded to or routed as appropriate.

Analysis Plan

The primary outcome will be the percentage of patients who complete the first dose of the COVID vaccine within 1 month of initial outreach. Secondary outcomes will be the completion of the first dose within 2 months, and completion of the vaccination process within 2 months of initial outreach. ~~We will also examine~~ **Additional outcomes will include the percentage of patients with invalid cell phone numbers (either wrong or non-textable numbers), non-response to text messaging,** the percentage of patients that are scheduled, text message responses (YES, INFO, NOT NOW, DONE), and number of phone calls made by the access center. **Heterogeneity of subgroup response through interaction analysis will be examined controlling for** ~~We will compare response rates by MPM~~ **patient portal**/email status, age, race/ethnicity, sex, insurance, and income (by zip code). **We will also examine vaccine completion by vaccine type, and code and examine non –keyword replies for themes.**

We estimate a 5% response rate to the phone call only. In order to minimize the outgoing phone call burden on the access center, we will include ~~1,000~~ **500** patients in the outbound phone call arm (Arm 1). For the scheduling flow comparison, we will allocate 10,000 patients in each text message arm (Arms 2-3). We will compare each text message arm to the phone call arm, resulting in 2 comparisons (arm 2 vs arm 1, arm 3 vs arm 1). Accounting for 2 pairwise comparisons with a P-value threshold of .025 (Bonferroni correction; .05/2), we estimate ~~91~~ **over 85**% power to detect a difference of ~~3~~ **4** percentage points (response rate of ~~8~~ **9**%) using the chi-squared test of proportions [STATA: power twoprop .05 .0~~8~~**9**, n1(~~1000~~ **500**) n2(10000) a(.025)].

For the messaging comparison, we will allocate ~~5,000~~ **2,500** patients in each arm. We will compare arms B-D to Arm A with 3 pairwise comparisons. Assuming a P-value threshold of .017 (.05/3) and a baseline response rate of 8% for text messaging, we estimate approximately 87% power to detect a difference of 2 percentage points (response rate of 10% for intervention arms) using the chi-squared test of proportions. [STATA: power twoprop .08 .10, n1(5000) n2 (5000) a(.017)]. We do not anticipate interaction between scheduling workflow and messaging arms.

Subject Confidentiality

Information about study subjects will be kept confidential and managed according to the requirements of the Health Insurance Portability and Accountability Act of 1996 (HIPAA). All PHI will be maintained on UPHS servers. Source documents are maintained in PennChart. No source documents will be printed or maintained in paper form at the study site. Data from PennChart will be recorded in Penn Medicine's REDCap system. The investigator and study team will have access to PHI within PennChart and REDCap. We will label all PHI within REDCap as identifiable information so that de-identified exports are possible. All reports that include identifiable information will be stored on the Innovation Center secure drive, maintained behind the UPHS firewall. Direct identifiers will be maintained on RedCap until manuscript publication in case additional chart review is needed for confirmation of results. Once data analysis and manuscripts have been published, direct identifiers will be deleted from RedCap and the de-identified database will be stored on the Innovation Center secure drive.

Sensitive Research Information

This Research does not involve collection of sensitive information about the subjects that should be excluded from the electronic medical record.

Subject Privacy

~~21,000~~ **20,500** patients will be identified through automated data extraction from the electronic health record (EHR). The research team will have no direct interaction with study participants. Text messaging will be conducted via the Way to Health platform. The initial text message will confirm identity prior to proceeding with additional text messaging for the intervention.

Data Disclosure

Patients **in Arm 2** who agree to scheduling an appointment for COVID-19 vaccination will receive up to 3 phone calls from Penn Medicine Access Center representatives in an attempt to schedule them for their appointment. As such, patients will be identified in EPIC via a scheduling queue for Access Center representative call queue purposes.

Consent

1. Consent Process

Overview

Waiver of consent for this study is requested. Please see below.

Children and Adolescents

Not applicable.

Adult Subjects Not Competent to Give Consent

Waiver of consent is being requested.

2. Waiver of Consent

Waiver or Alteration of Informed Consent*

Waiver or alteration of required elements of consent.

Minimal Risk*

This study involves no more than minimal risk to subjects. Outreach for COVID-19 vaccination is an operational imperative and this trial is embedded in routine clinical operations at Penn Medicine. All participants will receive messaging indicating they are eligible to receive the vaccine as well as a prompt for scheduling. The only research related activity is the randomization of patients to different types of scheduling and messaging strategies that will help inform best practices for future outreach to other patient populations. Participants must still consent to receive the vaccine at the time of vaccination, as is usual care.

Impact on Subject Rights and Welfare*

Subjects rights and welfare will not be adversely affected by the waiver of authorization and consent. All subjects will have the opportunity to voluntarily receive or not receive the COVID-19 vaccination. This trial aims to identify outreach approaches that might increase uptake.

Waiver Essential to Research*

We believe that we would not be able to practically conduct the research without waiver of consent. If we had to obtain either written or verbal consent ahead of time, it would substantially limit our study population and it may alter their participation in the intervention. Thus, we would only learn about the response rate for patients who we were able to speak to for consent. This would limit the generalizability to practice. We believe that subsequent completion of vaccination or deferral of participation in vaccination is still completely voluntary, and participants must provide consent at the time of vaccination. Obtaining waiver of consent would allow us to avoid the potential selection/volunteer bias for inclusion of patients particularly interested in vaccination that can occur when consent is required. Since our main objective is to understand the potential influence varying outreach strategies on participant behavior, we believe that obtaining consent would not be practicable. Additionally, we have received waiver of consent for similar studies related to population health outreach in the past.

Additional Information to Subjects

Participants who are vaccinated will be provided with appropriate information about possible side-effects and follow-up as per current vaccine guidelines and Penn Medicine standards of care. Participants will be offered their 2nd dose appointment as appropriate per the type of vaccine given at the first appointment.

Written Statement of Research*

We do not believe it would be reasonable to inform patients of their participation in this behavioral outreach study upon completion, as conveying the intent of the research (that it is not a vaccine trial) to a lay population would likely lead to unnecessary confusion. Calling all patients would be infeasible, and sending a text message would not be a sufficient platform to explain the nuances of the study. Text messaging might also crowd out other more important messaging from Penn Medicine about health promotion. As this is a behavior observation study and all participants will receive outreach, we feel the risk of harm from informing participants is greater than not informing them.

Risk / Benefit

Potential Study Risks

The risks associated with this study are no more than minimal. There is the potential risk of breach of confidentiality. We will minimize this risk by using de-identified information whenever possible and by maintaining all identifiable information on a secure drive and/or in a HIPAA-compliant system (e.g. REDCap) as is already being done through routine clinical operations. There is also the risk of physical discomfort from the vaccine. We will minimize this risk by communicating the potential discomforts at the time of the vaccination and by offering routine monitoring, as is the current standard practice for COVID-19 vaccinated patients at Penn Medicine.

Potential Study Benefits

If a participant completes their COVID-19 vaccination, they will potentially benefit by dramatically reducing the likelihood of infection by COVID-19 causing severe complications, hospitalization or death. Additionally, their vaccination will potentially benefit the population by increasing the overall rate of

immunity and slowing disease spread among those unable to receive the vaccine. Information learned from this study may additionally benefit society through a better understanding of how to effectively increase overall uptake of the COVID-19 vaccination, which could inform future vaccination campaigns or be applied to other population-wide contexts.

Alternatives to Participation (optional)
Participants may choose to opt-out of receiving text messaging and/or decline scheduling their vaccination.

Data and Safety Monitoring
Safety will be overseen by the PI and the study team. In the case of possible events, the PI or designee will review the study charts to evaluate events at each subject interaction to ensure the grade, relationship to the study procedure, expectedness, and the course of action for each subject is documented.

Risk / Benefit Assessment
The risks associated with this study are no more than minimal. Better knowledge of how to increase COVID-19 vaccine uptake could potentially address a major source of disparities among our patient population. Better vaccine uptake could potentially reduce the already increased disease burden on our most at-risk patients. For these reasons and those outlined in the above benefits section, the investigator believes that the risks of participating in the study are outweighed by the potential benefits of participating in the study.

Summary of Protocol Changes Modifications LOG

Protocol: Pragmatic Trial of COVID Vaccine Text Outreach Interventions
University of Pennsylvania Principal Investigator: Shivan Mehta, MD

Date of Submission	Description of Modification	Rationale for Modification	Approval date
03/22/2021	Initial submission		03/31/2021
04/28/2021	1) Updating study design:<br>- inclusion criteria<br>- study arm description<br>- sample size/power calculation<br>2) Texting content/messaging	1) Study Design:<br>a) Updated our inclusion criteria to match the current eligibility level of the city of Philadelphia, to include all resident adults (18+) who have been seen by a primary care physician at Penn Medicine in the last 5 years, regardless of risk.<br>b) Pivoted one study arm from calling patients back after they agree to scheduling and identify their preferred day and time frame, to offering patients the new in-bound Penn Medicine COVID-19 Vaccine Scheduling Hotline phone number<br>c) Reduced the number of patients in the Phone-Call Only arm from 1,000 to 500 to further accommodate call center activity<br>2) Texting Content/Messaging<br>a) Updated to reflect changes that were implemented as a result of the automated texting build process, as well as to accommodate the change in study arm and to provide additional clarity to recipients.	05/04/2021
07/07/2021	1) Updated additional outcomes to include:<br>- percentage of patients with invalid cell<br>- non-response to text messaging<br>- vaccine completion by vaccine type<br>- examination of non-keyword replies for thematic analysis		07/08/2021

11/10/2021	1) Updated inclusion criteria<br>- including future scheduled visits and clarifying PCP role<br>2) Updated analysis plan<br>- subgroup analysis		

Initial Statistical Analysis Plan

Analysis Plan

The primary outcome will be the percentage of patients who complete the first dose of the COVID vaccine within 1 month of initial outreach. Secondary outcomes will be the completion of the first dose within 2 months, and completion of the vaccination process within 2 months of initial outreach. We will also examine the percentage of patients that are scheduled, text message responses (YES, INFO, NOT NOW, DONE), and number of phone calls made by the access center. We will compare response rates by MPM/email status, age, race/ethnicity, sex, insurance, and income (by zip code).

We estimate a 5% response rate to the phone call only. In order to minimize the outgoing phone call burden on the access center, we will include 1,000 patients in the outbound phone call arm (Arm 1). For the scheduling flow comparison, we will allocate 10,000 patients in each text message arm (Arms 2-3). We will compare each text message arm to the phone call arm, resulting in 2 comparisons (arm 2 vs arm 1, arm 3 vs arm 1). Accounting for 2 pairwise comparisons with a P-value threshold of .025 (Bonferroni correction; .05/2), we estimate 91% power to detect a difference of 3 percentage points (response rate of 8%) using the chi-squared test of proportions [STATA: power twoprop .05 .08, n1(1000) n2(10000) a(.025)].

For the messaging comparison, we will allocate 5,000 patients in each arm. We will compare arms B-D to Arm A with 3 pairwise comparisons. Assuming a P-value threshold of .017 (.05/3) and a baseline response rate of 8% for text messaging, we estimate approximately 87% power to detect a difference of 2 percentage points (response rate of 10% for intervention arms) using the chi-squared test of proportions. [STATA: power twoprop .08 .10, n1(5000) n2 (5000) a(.017)]. We do not anticipate interaction between scheduling workflow and messaging arms.

Final Statistical Analysis Plan

****New changes from initial protocol notated in bold, parts removed from initial protocol notated in strikethrough**

Analysis Plan

The primary outcome will be the percentage of patients who complete the first dose of the COVID vaccine within 1 month of initial outreach. Secondary outcomes will be the completion of the first dose within 2 months, and completion of the vaccination process within 2 months of initial outreach. ~~We will also examine~~ **Additional outcomes will include the percentage of patients with invalid cell phone numbers (either wrong or non-textable numbers), non-response to text messaging,** the percentage of patients that are scheduled, text message responses (YES, INFO, NOT NOW, DONE), and number of phone calls made by the access center. **Heterogeneity of subgroup response through interaction analysis will be examined controlling for** ~~We will compare response rates by~~ ~~MPM~~ **patient portal**/email status, age, race/ethnicity, sex, insurance, and income (by zip code). **We will also examine vaccine completion by vaccine type, and code and examine non –keyword replies for themes.**

We estimate a 5% response rate to the phone call only. In order to minimize the outgoing phone call burden on the access center, we will include ~~1,000~~ **500** patients in the outbound phone call arm (Arm 1). For the scheduling flow comparison, we will allocate 10,000 patients in each text message arm (Arms 2-3). We will compare each text message arm to the phone call arm, resulting in 2 comparisons (arm 2 vs arm 1, arm 3 vs arm 1). Accounting for 2 pairwise comparisons with a P-value threshold of .025 (Bonferroni correction; .05/2), we estimate ~~91~~ **over 85**% power to detect a difference of ~~3~~ **4** percentage points (response rate of ~~8~~ **9**%) using the chi-squared test of proportions [STATA: power twoprop .05 .0~~8~~**9**, n1(~~1000~~ **500**) n2(10000) a(.025)].

For the messaging comparison, we will allocate ~~5,000~~ **2,500** patients in each arm. We will compare arms B-D to Arm A with 3 pairwise comparisons. Assuming a P-value threshold of .017 (.05/3) and a baseline response rate of 8% for text messaging, we estimate approximately 87% power to detect a difference of 2 percentage points (response rate of 10% for intervention arms) using the chi-squared test of proportions. [STATA: power twoprop .08 .10, n1(5000) n2 (5000) a(.017)]. We do not anticipate interaction between scheduling workflow and messaging arms.

Summary of Statistical Analysis Plan Modifications

7/7/21: Before beginning any analysis, we felt it necessary to revisit outcomes and provide a more detailed/thorough analysis plan to ensure both were as complete as possible.

11/9/21: Updated plan for subgroup analyses

Appendix A

Text Messaging Content

All patients in text messaging arms (2A-2D and 3A-3D) will receive the same introductory text and response options:

Intro/ID Confirmation Text:	Offer if Patient confirms ID:
Hello, this is Penn Medicine reaching out to you about your interest in the COVID-19 vaccine. Can you confirm you are [Patient Name]?<br><br>Please reply **YES** if this is correct or reply **NO** if we have reached the wrong number.<br><br>*You can reply STOP at any time to stop receiving these messages. Message & data rates may apply.<br><br>YES<br>NO	[Study Arm Message 2A-2D or 3A-3D] ... please reply **YES**.<br><br>If you have already scheduled or received your vaccine please reply, **DONE**<br><br>If you have questions you would like answered before scheduling your appointment please reply, **INFO**.<br><br>If you are not interested in scheduling your appointment at this time please reply, **NOT NOW**.<br><br>YES<br>DONE<br>INFO<br>NOT NOW

Patient replies **Y (YES)**: The reply to the patient after confirming their identification will vary by study arm, see Study Arm Offer Content below.

Patient replies **N (NO/Wrong Number)**: Our apologies, please disregard the previous message. Thank you.

<u>Study Arm Offer Content</u>

If patient replies **YES**, confirming their identity, they will receive an offer message according to scheduling type (Arm 2 or 3) and content type (A, B, C, or D).

[Arm 2 – Text + Outbound Call]

- **Arm 2A) Standard Messaging:** Our records show you are eligible for your COVID-19 vaccine at Penn Medicine. If you would like us to call you to schedule your appointment, please reply **YES**.

- **Arm 2B) Clinician Endorsement:** [Clinician name] recommends that you receive the COVID-19 vaccination. If you would like us to call you to schedule your appointment, please reply **YES.**

- **Arm 2C) Scarcity:** You have been selected to receive from the limited supply of COVID-19 vaccine at Penn Medicine. If you would like us to call you to schedule your appointment, please

reply **YES**.

- **Arm 2D) Opt-Out Frame:** We have reserved a COVID-19 vaccine appointment for you at Penn Medicine. If you would like to us to call to confirm, please reply **YES.**
    - Patient replies **YES** to any of the above: Great. One of our coordinators will be calling you within 1-2 business days to schedule your vaccine appointment at Penn Medicine.

[Arm 3 – Text + Inbound Call]

- **Arm 3A) Standard Messaging:** Our records show you are eligible for your COVID-19 vaccine at Penn Medicine. If you'd like to schedule your appointment, please reply **YES** to receive the number for the Penn Medicine Vaccine Scheduling Hotline.

- **Arm 3B) Clinician Endorsement:** [Clinician name] recommends that you receive the COVID-19 vaccination. If you'd like to schedule your appointment, please reply **YES** to receive the number for the Penn Medicine Vaccine Scheduling Hotline.

- **Arm 3C) Scarcity:** You have been selected to receive from the limited supply of COVID-19 vaccine at Penn Medicine. If you'd like to schedule your appointment, please reply **YES** to receive the number for the Penn Medicine Vaccine Scheduling Hotline.

- **Arm 3D) Opt-Out:** We have reserved a COVID-19 vaccine appointment for you at Penn Medicine. If you 'd like to schedule your appointment, please reply **YES** to receive the number for the Penn Medicine Vaccine Scheduling Hotline.
    - Patient replies **YES** to any of the above: Great! Please call 267-758-4902 within the next 7 days to schedule your vaccine appointment. The scheduling hotline operates Monday - Friday from 8am to 5pm.

After the **YES** response prompt, the same text will also include the following for all patients regardless of study arm:

If you have already scheduled or received your vaccine please reply, **DONE**

If you have questions you would like answered before scheduling your appointment please reply, **INFO**.

If you are not interested in scheduling your appointment at this time please reply, **NOT NOW**.

- Patient replies **DONE**: Thank you.

- Patient replies **INFO**: You can find a list of common questions and answers here, bit.ly/vaccine. You can also reply to this message with your questions and we will try to get back to you with answers. If you are ready to move forward with scheduling your appointment please reply, **CALL ME** within the next 3 days.
    - Any response: Thank you. Our team will get back to you and try to answer your question. You can reply **CALL ME** within the next 3 days to proceed with scheduling your

vaccine appointment.

- Patient replies **NOT NOW**: Thank you. Could you please tell us why you are not currently interested in scheduling your vaccine? If you change your mind and are ready to move forward with scheduling your appointment please reply, **CALL ME**, within the next 3 days.
    - Any response: Thank you. We appreciate you sharing your perspective. You can reply **CALL ME** within the next 3 days to proceed with scheduling your vaccine appointment.
- Patient replies **CALL ME:** Great. One of our coordinators will be calling you within 1-2 business days to schedule your vaccine appointment at Penn Medicine.

Non-Response:

If no response received to either ID Confirmation or Offer [+4 days]: We haven't heard back from you. Please respond to the question above.

If no response received to either ID Confirmation or Offer [+9 days]: We haven't heard back from you. Please respond to the question above.
