# Supplementary material for: Effect of Text Messaging and Behavioral Interventions on COVID-19 Vaccination Uptake: A Randomized Clinical Trial
Source: JAMA Netw Open. 2022 Jun 13;5(6):e2216649. doi: 10.1001/jamanetworkopen.2022.16649 (PMC9194662; doi:10.1001/jamanetworkopen.2022.16649)
Supplement: Supplement 2. — eTable 1. Vaccine Received Prior to Outreach eTable 2. Dose 1 Completion in One Month by Subgroup (Scheduling Modality) eTable 3. Dose 1 Completion in One Month by Subgroup (Messaging Content) eTable 4. Final Text Response [file jamanetwopen-e2216649-s002.pdf]

Supplementary Online Content

Mehta SJ, Mallozzi C, Shaw PA, et al. Effect of text messaging and behavioral interventions on COVID-19 vaccination uptake: a randomized clinical trial. *JAMA Netw Open.* 2022;5(6):e2216649. doi:10.1001/jamanetworkopen.2022.16649

eTable 1. Vaccine Received Prior to Outreach
eTable 2. Dose 1 Completion in One Month by Subgroup (Scheduling Modality)
eTable 3. Dose 1 Completion in One Month by Subgroup (Messaging Content)
eTable 4. Final Text Response

This supplementary material has been provided by the authors to give readers additional information about their work.

© 2022 Mehta SJ et al. *JAMA Network Open.*

eTable 1. Vaccine Received Prior to Outreach

Study Group	Outbound Phone Only	Text + Outbound Call				Text + Inbound Call				Total
		Standard Messaging	Clinician Endorsement	Scarcity	Endowment	Standard Messaging	Clinician Endorsement	Scarcity	Endowment	
**N**	472	2,393	2,389	2,392	2,385	2,380	2,382	2,383	2,378	19,554
**Rcv'd 1 Dose Prior, N (%)**	82 (17.4)	422 (17.6)	404 (16.9)	415 (17.4)	428 (18.0)	462 (19.4)	447 (18.8)	449 (18.8)	400 (16.8)	3,509 (18.0)
**Rcv'd Full Series[1] Prior, N (%)**	52 (11.0)	279 (11.7)	262 (11.0)	278 (11.6)	273 (11.5)	327 (13.7)	274 (11.5)	315 (13.2)	255 (10.7)	2,315 (11.8)

[1] All patients that received the full series are included in the group that received 1 dose.

© 2022 Mehta SJ et al. *JAMA Network Open.*

eTable 2. Dose 1 Completion in One Month by Subgroup (Scheduling Modality)

	Outbound Phone Only N (%)	Text + Outbound Call N (%)	Text + Inbound Call N (%)	Treatment Effect Size Log OR (95% CI) (Text + Outbound Call v Outbound Call Only)	Treatment Effect Size Log OR (95% CI) (Text + Inbound Call v Outbound Call Only)
**Overall**	14/390	243/7,890	253/7,765	-0.14 (-0.69 – 0.41)	-0.07 (-0.62 – 0.49)
**Sex**					
Male	7/155 (4.5)	104/3,237 (3.2)	94/3,235 (2.9)	-0.38 (-1.73 – 0.97)	-0.47 (-1.81 – 0.88)
Female	7/235 (3.0)	139/4,653 (3.0)	159/4,530 (3.5)	0.05 (-0.71 – 0.82)	0.23 (-0.54 – 0.99)
**Race**					
White	2/189 (1.1)	87/4,117 (2.1)	73/4,039 (1.8)	0.43 (-0.67 – 1.53)	0.27 (-0.83 – 1.37)
Black	8/127 (6.3)	117/2,338 (5.0)	137/2,241 (6.1)	-0.26 (-2.00 – 1.47)	-0.05 (-1.78 – 1.69)
Asian/Pacific Islander	3/26 (11.5)	18/600 (3.0)	17/614 (2.8)	-1.46 (-3.45 – 0.53)	-1.48 (-3.48 – 0.51)
Other[1]	1/48 (2.1)	21/835 (2.5)	26/871 (3.0)	0.74 (-2.70 – 4.18)	0.27 (-0.83 – 1.37)
**Ethnicity**					
Hispanic	1/26 (3.9)	19/486 (3.9)	21/455 (4.6)	0.21 (-2.48 – 2.91)	0.34 (-2.35 – 3.03)
Not-Hispanic	13/352 (3.7)	217/7,238 (3.0)	226/7,136 (3.2)	-0.20 (-4.05 – 3.66)	-0.12 (-3.97 – 3.72)
Other[2]	0/12 (0.0)	7/166 (4.2)	6/174 (3.4)	1.49 (-2.50 – 5.47)	1.32 (-2.67 – 5.31)
**Age Category**					
18-29	7/129 (5.4)	72/2,162 (3.3)	72/2,187 (3.3)	-0.35 (-1.22 – 0.52)	-0.36 (-1.23 – 0.51)
30-39	2/127 (1.6)	74/3,129 (2.4)	65/3,034 (2.1)	0.27 (-1.50 – 2.04)	0.16 (-1.61 – 1.93)
40-49	2/73 (2.7)	48/1,386 (4.2)	56/1,379 (4.1)	0.03 (-1.77 – 1.82)	0.24 (-1.55 – 2.03)
50-64	3/60 (5.0)	48/1,143 (4.2)	57/1,109 (5.1)	-0.21 (-1.94 – 1.51)	0.00 (-1.72 – 1.71)
65+	0/1 (0.0)	1/70 (1.4)	3/56 (5.4)	-1.32 (-4.60 – 1.95)	0.54 (-2.22 – 3.30)
**Income Quartile[3]**					
< $35,000 / Unknown[4]	7/112 (6.3)	88/2,180 (4.0)	100/2,104 (4.8)	-0.49 (-1.27 – 0.29)	-0.28 (-1.05 – 0.50)
$35,000 – 59,999	6/92 (6.5)	81/1,999 (4.1)	82/1,959 (4.2)	-0.48 (-1.90 – 0.94)	-0.46 (-1.87 – 0.96)
$60,000 – 76,099	1/68 (1.5)	33/1,507 (2.2)	29/1,517 (1.9)	0.76 (-1.98 – 3.51)	0.60 (-2.14 – 3.34)
≥ $76,100	0/118 (0.0)	41/2,204 (1.9)	42/2,185 (1.9)	3.82 (2.66 – 4.98)	3.85 (2.69 – 5.01)
**Patient Portal Status**					
Active	12/308 (3.9)	206/6,419 (3.2)	207/6,335 (3.3)	-0.38 (-1.73 – 0.97)	-0.47 (-1.81 – 0.88)
Inactive	2/82 (2.4)	37/1,471 (2.5)	46/1,430 (3.2)	-0.04 (-1.49 – 1.40)	0.25 (-1.19 – 1.69)
**Insurance Coverage Type**					
Commercial	9/308 (2.9)	176/6,309 (2.8)	176/6,252 (2.8)	0.02 (-0.68 – 0.71)	0.02 (-0.67 – 0.71)
Medicaid	4/59 (6.8)	51/1,135 (4.5)	63/1,089 (5.8)	-0.57 (-1.99 – 0.85)	-0.27 (-1.68 – 1.15)
Medicare	0/5 (0)	10/138 (7.2)	7/112 (6.3)	0.61 (-0.92 – 2.14)	0.53 (-1.06 – 2.13)
Other[5]	1/18 (5.6)	6/308 (1.9)	7/312 (2.2)	-0.56 (-3.72 – 2.60)	-0.39 (-3.53 – 2.75)
**Last Patient Visit**					
<= 2 Years	12/316 (3.8)	227/6,456 (3.5)	242/6,336 (3.8)	-0.05 (-2.35 – 2.25)	0.05 (-2.29 – 2.38)
> 2 Years	2/74 (2.7)	16/1,431 (1.1)	11/1,429 (0.8)	-0.80 (-2.37 – 0.77)	-1.17 (-2.77 – 0.43)

[1] Other was combined with American Indian, Unknown, and Refused due to very small sample size
[2] Other was combined with Unknown and Refused due to very small sample size
[3] American Community Survey (2015-2019) Median Household Income in 2019 inflation adjusted dollars
[4] Missing/Unknown was combined with <$35,000 due to very small sample size
[5]Other was combined with No Insurance and Unknown due to very small sample size

© 2022 Mehta SJ et al. *JAMA Network Open.*

eTable 3. Dose 1 Completion in One Month by Subgroup (Messaging Content)

	Standard Msg, N (%)	Clinician Endorse, N (%)	Scarcity, N (%)	Endowment, N (%)	Treatment Effect Size Log OR (95% CI) (Endorsement v Standard Msg)	Treatment Effect Size Log OR (95% CI) (Scarcity v Standard Msg)	Treatment Effect Size Log OR (95% CI) (Endowment v Standard Msg)
**Sex**							
Male	43/1,611 (2.7)	54/1,622 (3.3)	48/1,575 (3.1)	53/1,664 (3.2)	0.20 (-0.42 – 0.81)	0.07 (-0.59 – 0.73)	0.16 (-0.44 – 0.77)
Female	75/2,278 (3.3)	81/2,298 (3.5)	52/2,336 (2.2)	90/2,271 (4.0)	0.11 (-0.22 – 0.43)	-0.38 (-0.74 - -0.02)	0.19 (-0.12 – 0.51)
**Race**							
White	49/2,031 (2.4)	44/2,041 (2.2)	29/2,032 (1.4)	38/2,052 (1.9)	-0.13 (-0.55 – 0.28)	-0.53 (-1.00 - -0.07)	-0.30 (-0.73 – 0.13)
Black	49/1,131 (4.3)	73 /1,147 (6.4)	53/1,151 (4.6)	79/1,150 (6.9)	0.44 (-0.25 – 1.13)	0.06 (-0.71 – 0.83)	0.50 (-0.21 – 1.21)
Asian/Pacific Islander	8/295 (2.7)	12/314 (3.8)	6/298 (2.0)	9/307 (2.9)	0.31 (-0.77 – 1.39)	-0.37 (-1.62 – 0.88)	0.05 (-1.08 – 1.19)
Other[1]	12/432 (2.8)	6/418 (1.4)	12/430 (2.8)	17/426 (4.0)	-0.65 (-1.81 – 0.50)	-0.06 (-1.10 – 0.99)	0.34 (-0.63 – 1.31)
**Ethnicity**							
Hispanic	13/234 (5.6)	15/245 (6.1)	2/229 (0.9)	10/233 (4.3)	0.21 (-0.59 – 1.00)	-1.98 (-3.58 - -0.39)	-0.31 (-1.18 – 0.55)
Not-Hispanic	103/3,566 (2.9)	119/3,585 (3.3)	94/3,608 (2.6)	127/3,615 (3.5)	0.15 (-1.01 – 1.30)	-0.12 (-2.40 – 2.16)	0.21 (-1.05 – 1.46)
Other[2]	2/89 (2.2)	1/90 (1.1)	4/74 (5.4)	6/87 (6.9)	-0.88 (-3.57 – 1.82)	0.79 (-1.90 – 3.48)	0.85 (-1.11 – 2.80)
**Age**							
18-29	36/1,080 (3.3)	38/1,019 (3.7)	28/1,123 (2.5)	42/1,127 (3.7)	0.14 (-0.32 – 0.61)	-0.28 (-0.79 – 0.23)	0.09 (-0.37 – 0.43)
30-39	34/1,565 (2.2)	39/1,587 (2.5)	29/1,520 (1.9)	37/1,491 (2.5)	0.12 (-0.69 – 0.93)	-0.15 (-1.03 – 0.73)	0.13 (-0.68 – 0.94)
40-49	22/673 (3.3)	30/718 (4.2)	26/669 (3.9)	26/705 (3.7)	0.25 (-0.61 – 1.12)	0.17 (-0.75 – 1.10)	0.15 (-0.73 – 1.02)
50-64	25/541 (4.6)	28/571 (4.9)	17/565 (3.0)	35/575 (6.1)	0.11 (-0.75 – 0.97)	-0.48 (-1.44 – 0.48)	0.35 (-0.49 – 1.19)
65+	1/30 (3.3)	0/25 (0.0)	0/34 (0.0)	3/37 (8.1)	-7.0 (-9.26 - -4.75)	-6.27 (-8.53 - -4.02)	0.56 (-1.97 – 3.10)
**Income[3]**							
< $35,000 / Unknown[4]	46/1,056 (4.4)	54/1,090 (5.0)	34/1,080 (3.1)	54/1,058 (5.1)	0.15 (-0.26 – 0.55)	-0.35 (-0.80 – 0.10)	0.15 (-0.25 – 0.56)
$35,000 – 59,999	38/984 (3.9)	36/951 (3.8)	38/1,007 (3.8)	51/1,016 (5.0)	-0.01 (-0.75 – 0.73)	-0.03 (-0.83 - 0.76)	0.27 (-0.45 – 0.99)
$60,000 – 76,099	20/765 (2.6)	18/758 (2.4)	13/740 (1.8)	11/761 (1.5)	-0.14 (-1.00 – 0.73)	-0.41 (-1.36 – 0.55)	-0.61 (-1.55 – 0.33)
≥ $76,100	14/1,084 (1.3)	27/1,121 (2.4)	15/1,084 (1.4)	27/1,100 (2.5)	0.66 (-0.22 – 1.54)	0.08 (-0.91 – 1.06)	0.66 (-0.22 – 1.54)
**Patient Portal Status**							
Active	99/3,134 (3.2)	112/3,200 (3.5)	83/3,162 (2.6)	119/3,258 (3.7)	0.11 (-0.81 – 1.04)	-0.14 (-0.80 – 0.53)	0.15 (-0.76 – 1.07)
Inactive	19/755 (2.5)	23/720 (3.2)	17/749 (2.3)	24/677 (3.6)	0.27 (-0.36 – 0.89)	-0.20 (-1.19 – 0.79)	0.31 (-0.31 – 0.92)
**Insurance Coverage Type**							
Commercial	79/3,149 (2.5)	98/3,133 (3.1)	68/3,131 (2.2)	107/3,148 (3.4)	0.23 (-0.08 – 0.53)	-0.16 (-0.49 – 0.17)	0.30 (0.00 – 0.59)
Medicaid	27/548 (4.9)	32/537 (6.0)	27/569 (4.7)	28/570 (4.9)	0.23 (-0.46 – 0.91)	-0.04 (-0.77 – 0.68)	0.03 (-0.66 – 0.73)
Medicare	7/54 (12.9)	2/70 (2.9)	3/62 (4.8)	5/64 (7.8)	-1.65 (-3.42 – 0.11)	-1.06 (-2.57 – 0.46)	-0.56 (-1.86 – 0.73)
Other[5]	5/138 (3.6)	3/180 (1.7)	2/149 (1.3)	3/153 (2.0)	-0.67 (-2.20 – 0.86)	-0.92 (-2.61 – 0.77)	-0.57 (-2.11 – 0.97)
**Last Patient Visit**							
<= 2 Years	107/3,184 (3.4)	128/3,182 (4.0)	96/3,210 (3.0)	138/3,219 (4.3)	0.19 (-1.17 – 1.56)	-0.13 (-1.79 (1.52)	0.25 (-1.29 (1.79)
> 2 Years	11/705 (1.6)	7/738 (0.9)	4/701 (0.6)	5/716 (0.7)	-0.50 (-1.45 – 0.45)	-1.00 (-2.15 – 0.16)	-0.85 (-1.92 – 0.22)

[1] Other was combined with American Indian, Unknown, and Refused due to very small sample size
[2] Other was combined with Unknown and Refused due to very small sample size
[3] American Community Survey (2015-2019) Median Household Income in 2019 inflation adjusted dollars
[4] Missing/Unknown was combined with <$35,000 due to very small sample size
[5] Other was combined with No Insurance and Unknown due to very small sample size

© 2022 Mehta SJ et al. *JAMA Network Open.*

eTable 4. Final Text Response

Outcome	Text + Outbound Call				Text + Inbound Call				Total (n=15,655)
	Standard Msg (n=1,971)	Clinician Endorse (n=1,985)	Scarcity (n=1,977)	Endowment (n=1,957)	Standard Msg (n=1,918)	Clinician Endorse (n=1,935)	Scarcity (n=1,934)	Endowment (n=1,978)	
**Message Fail, N (%)**	87 (4.4)	100 (5.0)	84 (4.3)	101 (5.2)	90 (4.7)	86 (4.4)	76 (3.9)	99 (5.0)	723 (4.6)
**No Response ID, N (%)**	670 (34.0)	625 (31.5)	636 (32.2)	646 (33.0)	661 (34.5)	594 (30.7)	666 (34.4)	620(31.3)	5,118 (32.7)
**No Response Offer, N (%)**	60 (3.0)	54 (2.7)	55 (2.8)	51 (2.6)	64 (3.3)	48 (2.5)	53 (2.7)	41 (2.1)	426 (2.7)
**Yes, N (%)**	47 (2.4)	51 (2.6)	55 (2.8)	52 (2.7)	68 (3.6)	58 (3.0)	58 (3.0)	69 (3.5)	458 (2.9)
**Info, N (%)**	12 (0.6)	19 (1.0)	19 (1.0)	23 (1.2)	9 (0.5)	17 (0.9)	14 (0.7)	18 (0.9)	131 (0.8)
**Not Now, N (%)**	85 (4.3)	84 (4.2)	74 (3.7)	86 (4.4)	68 (3.6)	75 (3.9)	68 (3.5)	87 (4.4)	627 (4.0)
**Done, N (%)**	435 (22.1)	480 (24.2)	467 (23.6)	445 (22.7)	445 (23.2)	486 (25.1)	435 (22.5)	468 (23.7)	3,661 (23.4)
**Call Me,[1] N (%)**	6 (0.3)	3 (0.2)	4 (0.2)	6 (0.3)	0 (0.0)	4 (0.2)	5 (0.3)	5 (0.3)	33 (0.2)
**Stop, N (%)**	416 (21.1)	447 (22.5)	423 (21.4)	401 (20.5)	387 (20.2)	422 (21.8)	407 (21.0)	399 (20.2)	3,302 (21.1)
**Unstop, N (%)**	8 (0.4)	4 (0.2)	11 (0.6)	7 (0.4)	5 (0.3)	10 (0.5)	9 (0.5)	0 (0.0)	54 (0.3)
**Wrong Patient, N (%)**	138 (7.0)	113 (5.7)	139 (7.0)	121 (6.2)	111 (5.8)	128 (6.6)	133 (6.9)	158 (8.0)	1,041 (6.7)
**Recode Already Vaccinated,[2] N (%)**	7 (0.4)	5 (0.3)	10 (0.5)	18 (0.9)	10 (0.5)	7 (0.4)	10 (0.5)	14 (0.7)	81 (0.5)

[1] Patients who texted 'Not Now' or 'Info' were given an option to reply 'Call Me' if they decided to schedule a vaccine appointment at a later date.

[2] Patients who did not reply DONE but who otherwise indicated by free text they had completed vaccination were recoded as "Already Vaccinated.".

© 2022 Mehta SJ et al. *JAMA Network Open.*
