# Supplementary material for: Effect of Text Messaging and Behavioral Interventions on COVID-19 Vaccination Uptake: A Randomized Clinical Trial
Source: JAMA Netw Open. 2022 Jun 13;5(6):e2216649. doi: 10.1001/jamanetworkopen.2022.16649 (PMC9194662; doi:10.1001/jamanetworkopen.2022.16649)
Supplement: Supplement 3. — Data Sharing Statement [file jamanetwopen-e2216649-s00.pdf]

Data Sharing Statement

Mehta. Effect of Text Messaging and Behavioral Interventions on COVID-19 Vaccination Uptake. *JAMA Netw Open*. Published June 13, 2022.
doi:10.1001/jamanetworkopen.2022.16649

Data
Data available: No

Additional Information
Explanation for why data not available: We do not have health system and IRB approval
